# Supplementary figures and images for: Heat stress-induced activation of MAPK pathway attenuates Atf1-dependent epigenetic inheritance of heterochromatin in fission yeast
Source: eLife. 2024 Jan 30;13:e90525. doi: 10.7554/eLife.90525 (PMC10863984; doi:10.7554/eLife.90525)

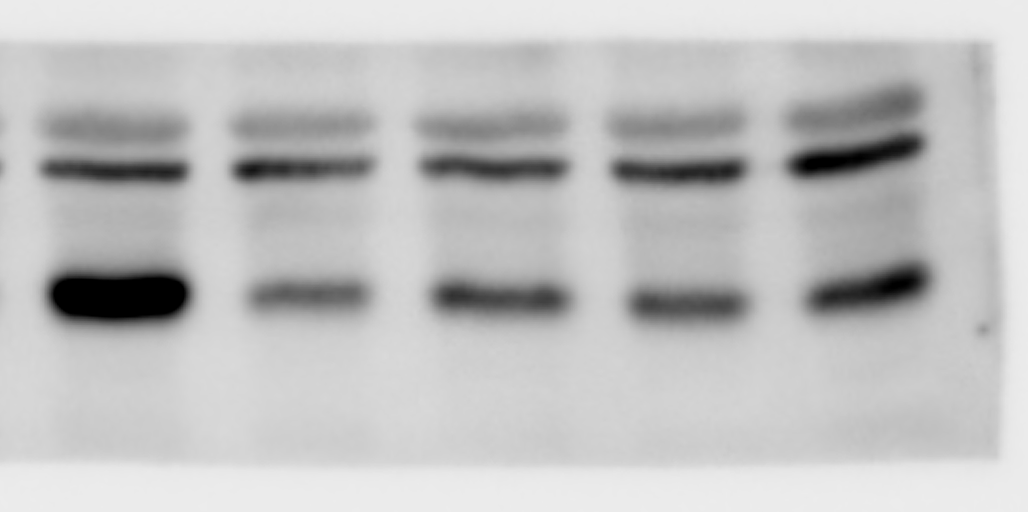

Supplement: Figure 1—figure supplement 2—source data 2. [file elife-90525-fig1-figsupp2-data2.zip › Figure 1-figure supplement 2-Source Data 2 [full raw unedited blot (mat3M-GFP) for Figure B].tif]

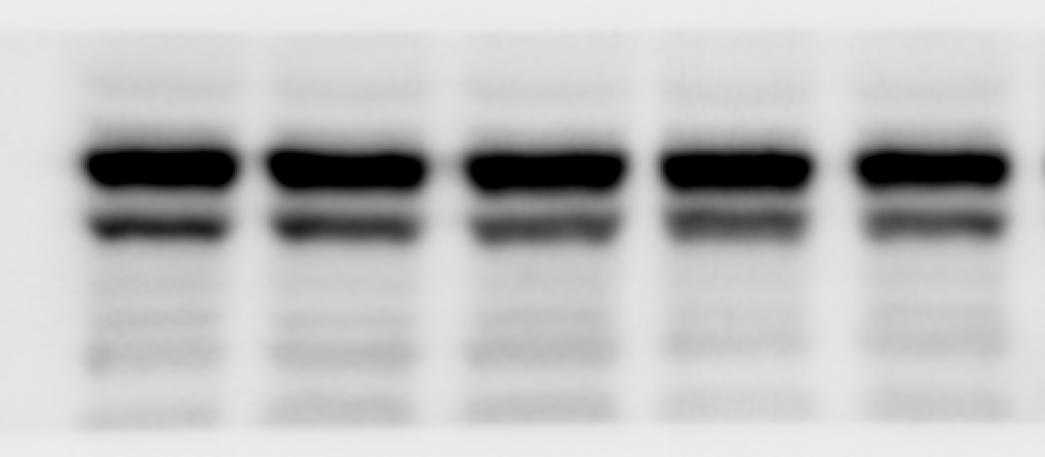

Supplement: Figure 1—figure supplement 2—source data 3. [file elife-90525-fig1-figsupp2-data3.zip › Figure 1-figure supplement 2-Source Data 3 [full raw unedited blot (Cdc2) for Figure B].tif]

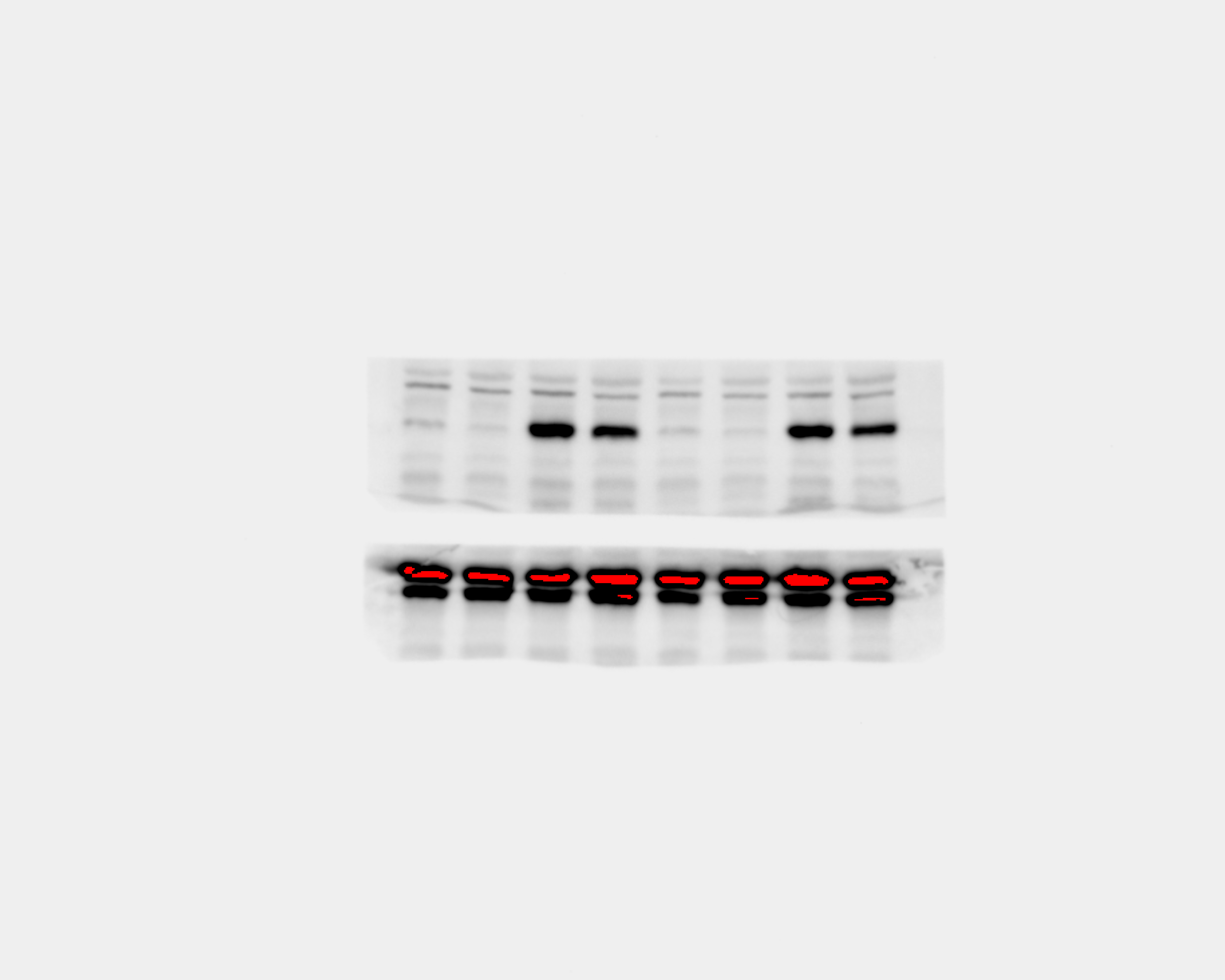

Supplement: Figure 1—figure supplement 2—source data 4. [file elife-90525-fig1-figsupp2-data4.zip › Figure 1-figure supplement 2-Source Data 4 [full raw unedited blot (imr1R-GFP) for Figure B].tif]

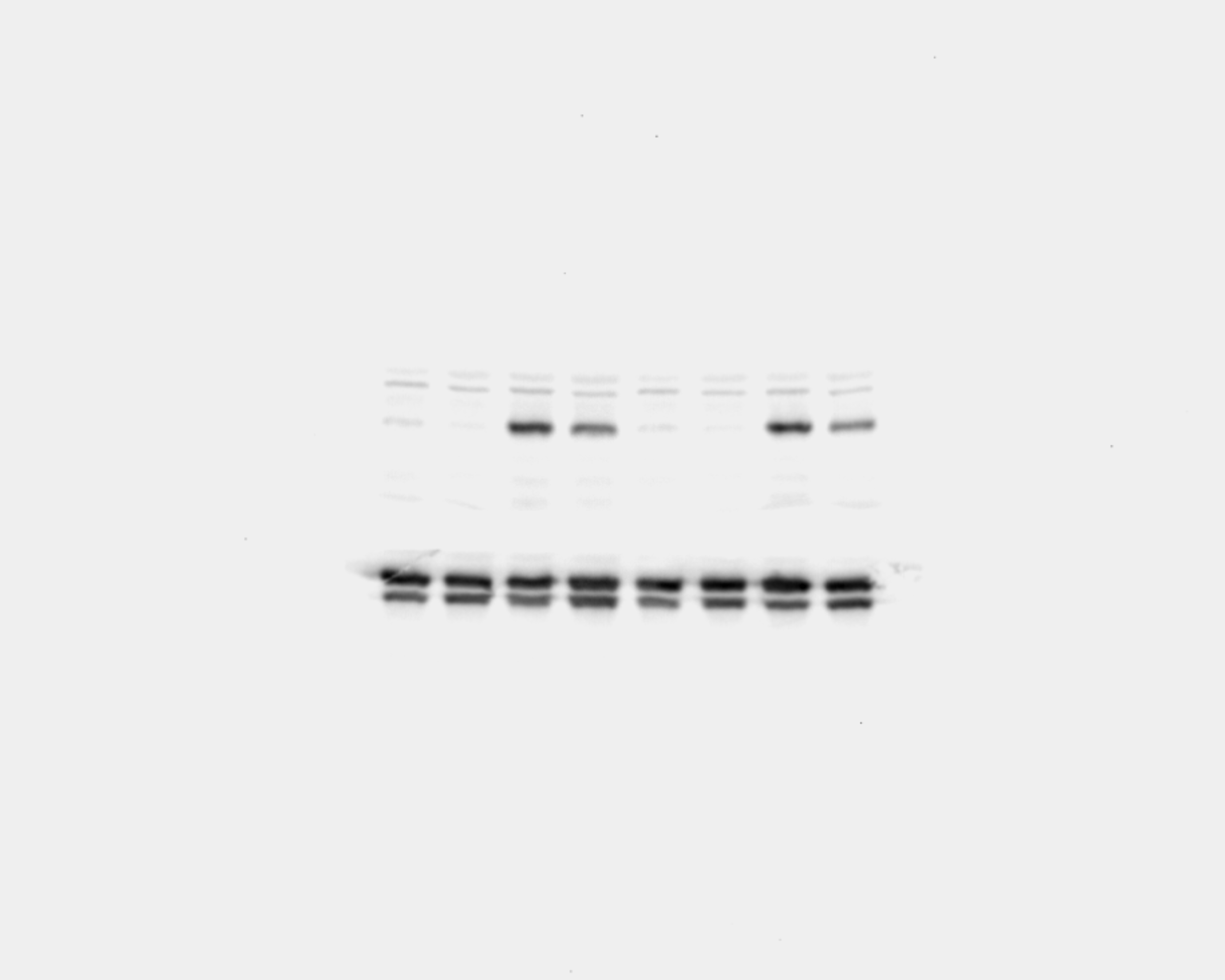

Supplement: Figure 1—figure supplement 2—source data 5. [file elife-90525-fig1-figsupp2-data5.zip › Figure 1-figure supplement 2-Source Data 5 [full raw unedited blot (Cdc2) for Figure B].tif]

Figure 1-figure supplement 2.

**B**

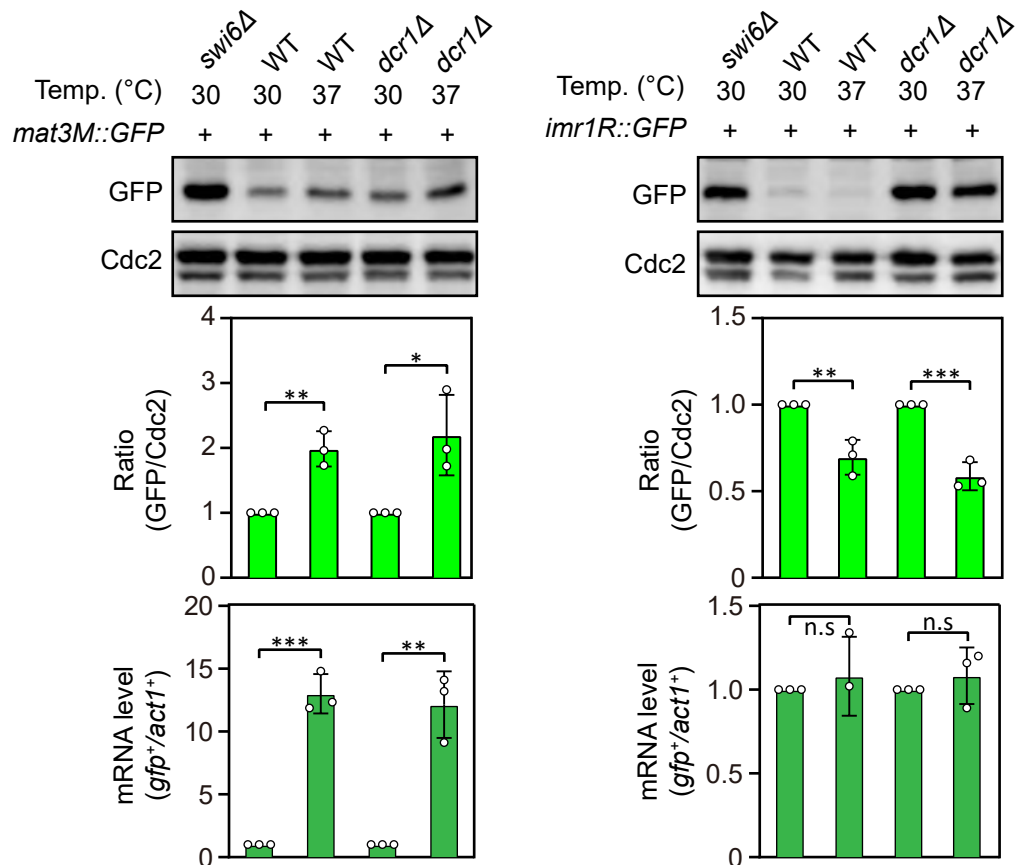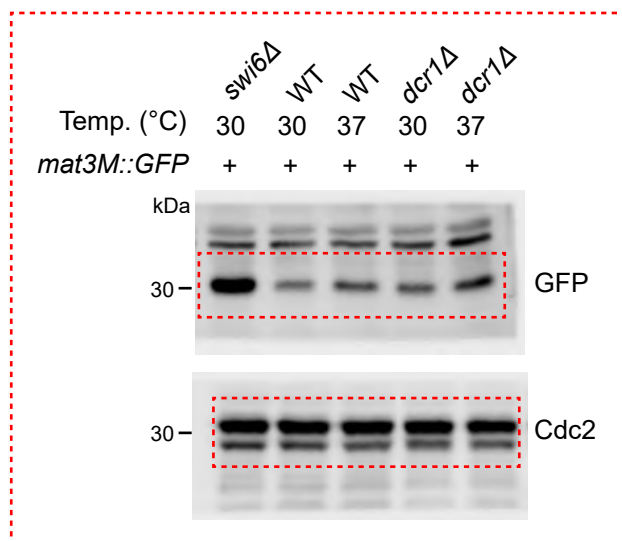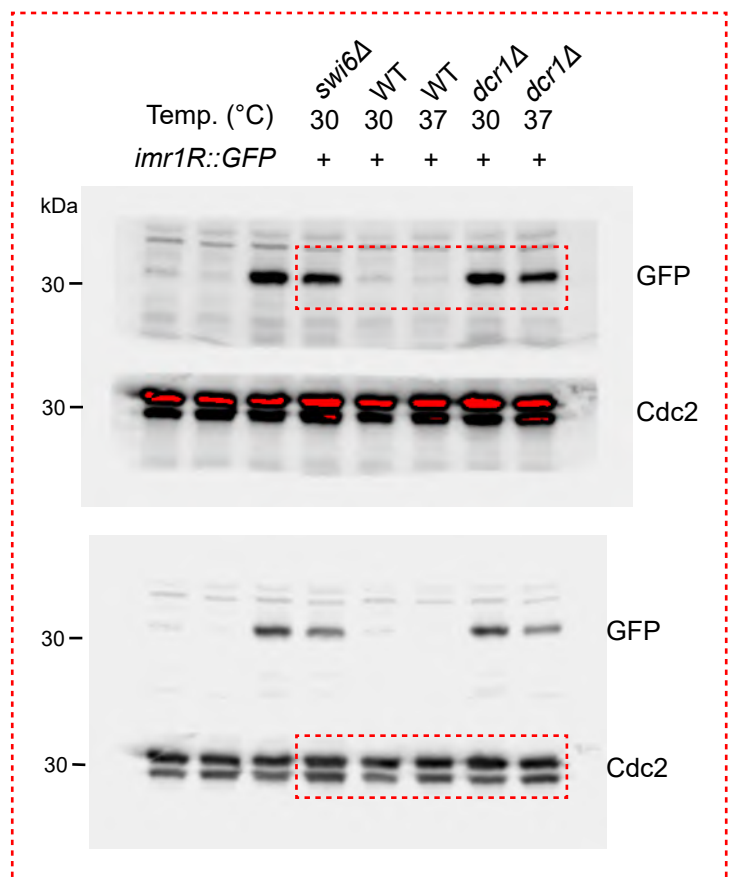

Supplement: Figure 1—figure supplement 2—source data 6. [file elife-90525-fig1-figsupp2-data6.pdf]

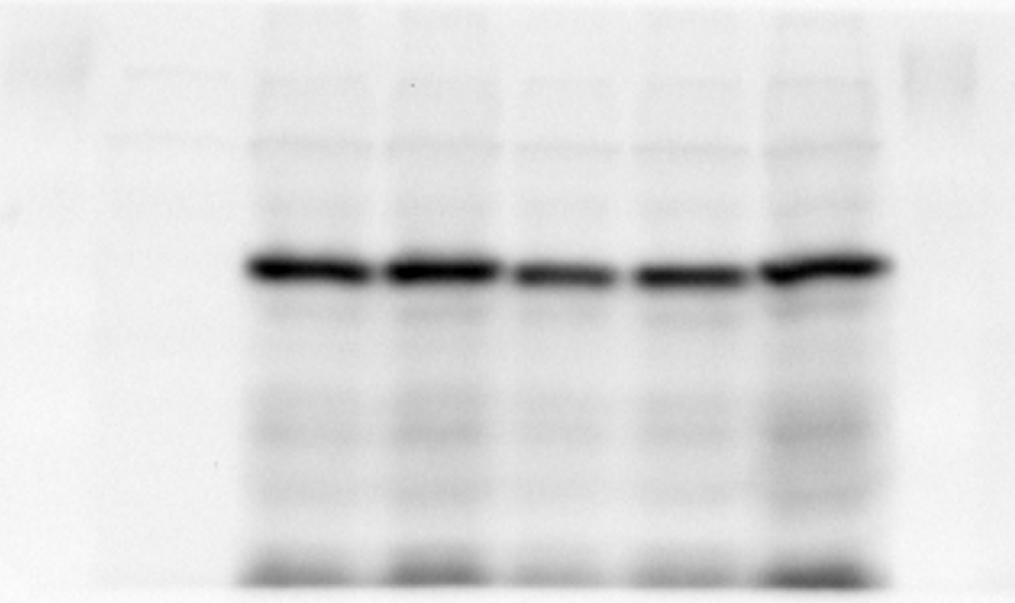

Supplement: Figure 3—source data 2. [file elife-90525-fig3-data2.zip › Figure 3-Source Data 2 [full raw unedited blot (Atf1) of Figure3C].tif]

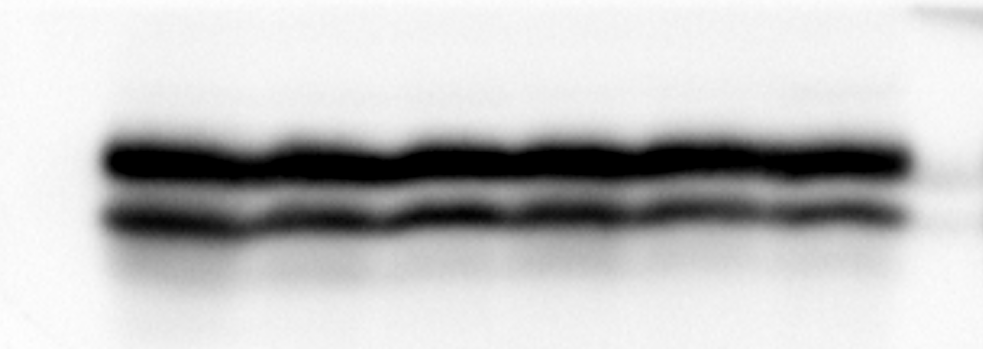

Supplement: Figure 3—source data 3. [file elife-90525-fig3-data3.zip › Figure 3-Source Data 3 [full raw unedited blot (Cdc2) of Figure3C].tif]

C

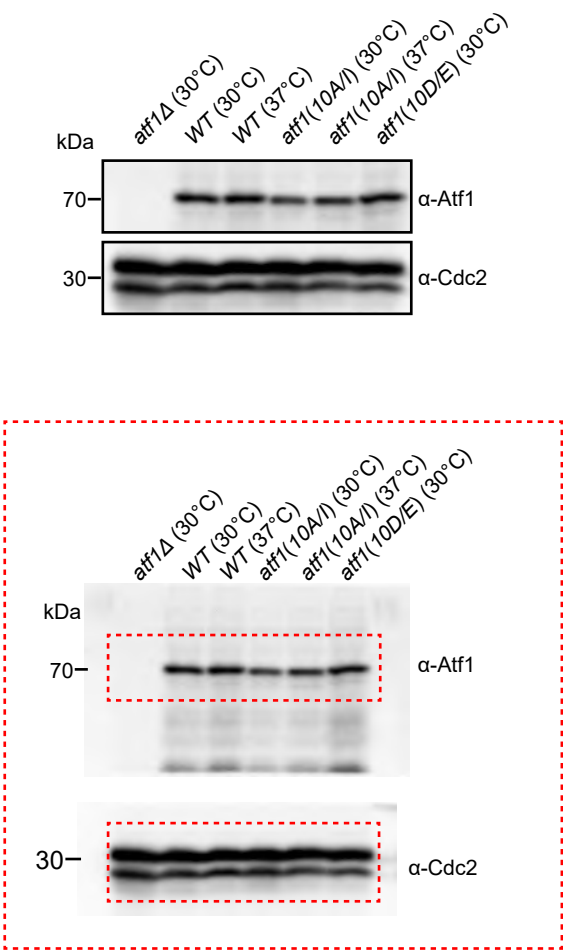

Supplement: Figure 3—source data 4. [file elife-90525-fig3-data4.pdf]

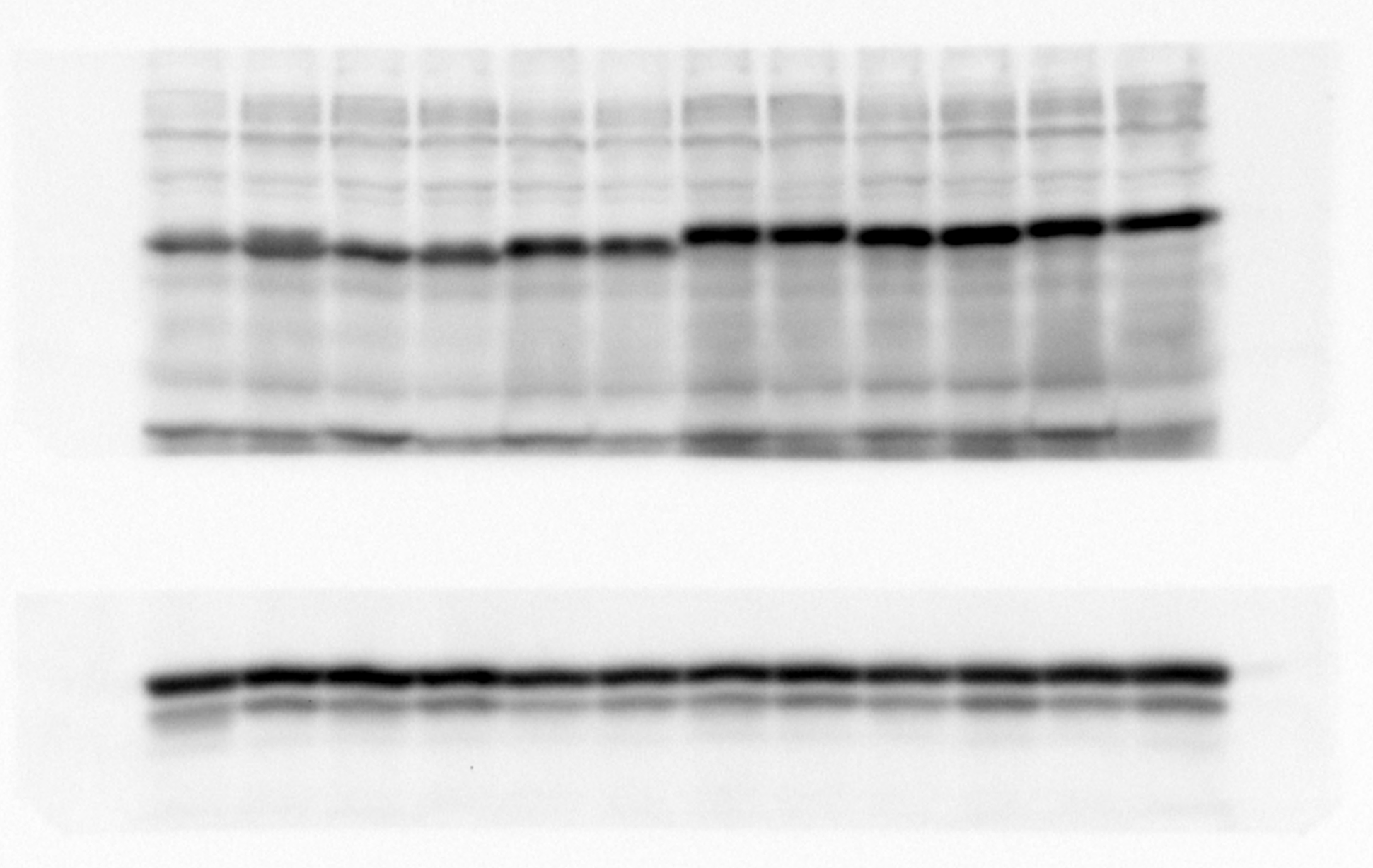

Supplement: Figure 3—figure supplement 2—source data 1. [file elife-90525-fig3-figsupp2-data1.zip › Figure 3-figure supplement 2-Source Data 1 [full raw unedited blots (Atf1 and Cdc2) for Figure C].tif]

Figure 3-figure supplement 2C

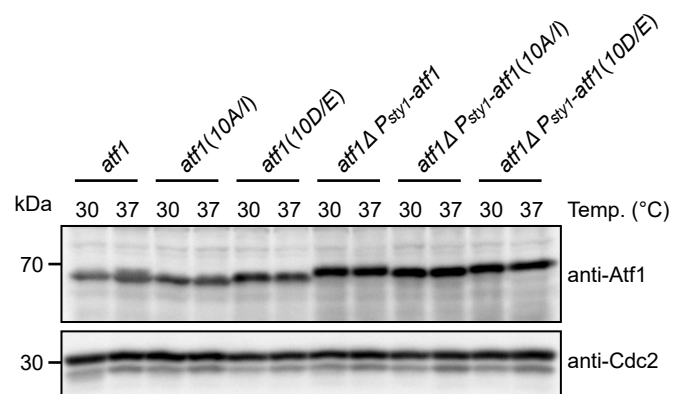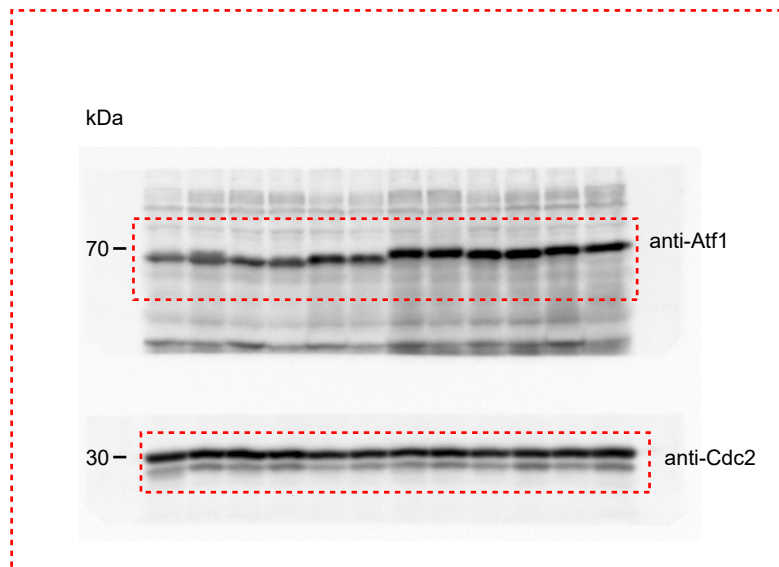

Supplement: Figure 3—figure supplement 2—source data 2. [file elife-90525-fig3-figsupp2-data2.pdf]

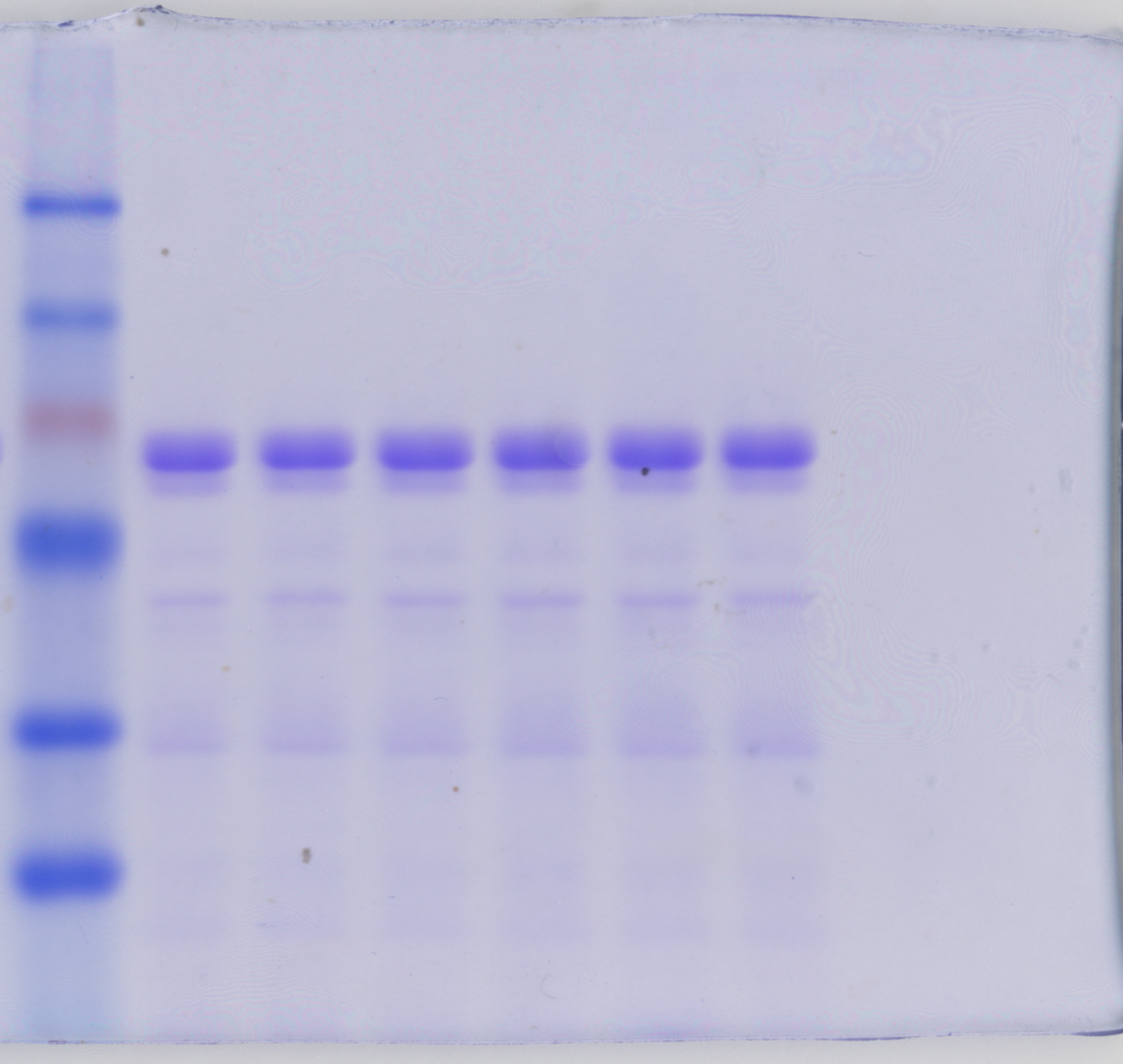

Supplement: Figure 4—source data 2. [file elife-90525-fig4-data2.zip › Figure 4-Source Data 2 [full raw unedited Coomassie gel (His-Swi6) of Figure 4A].tif]

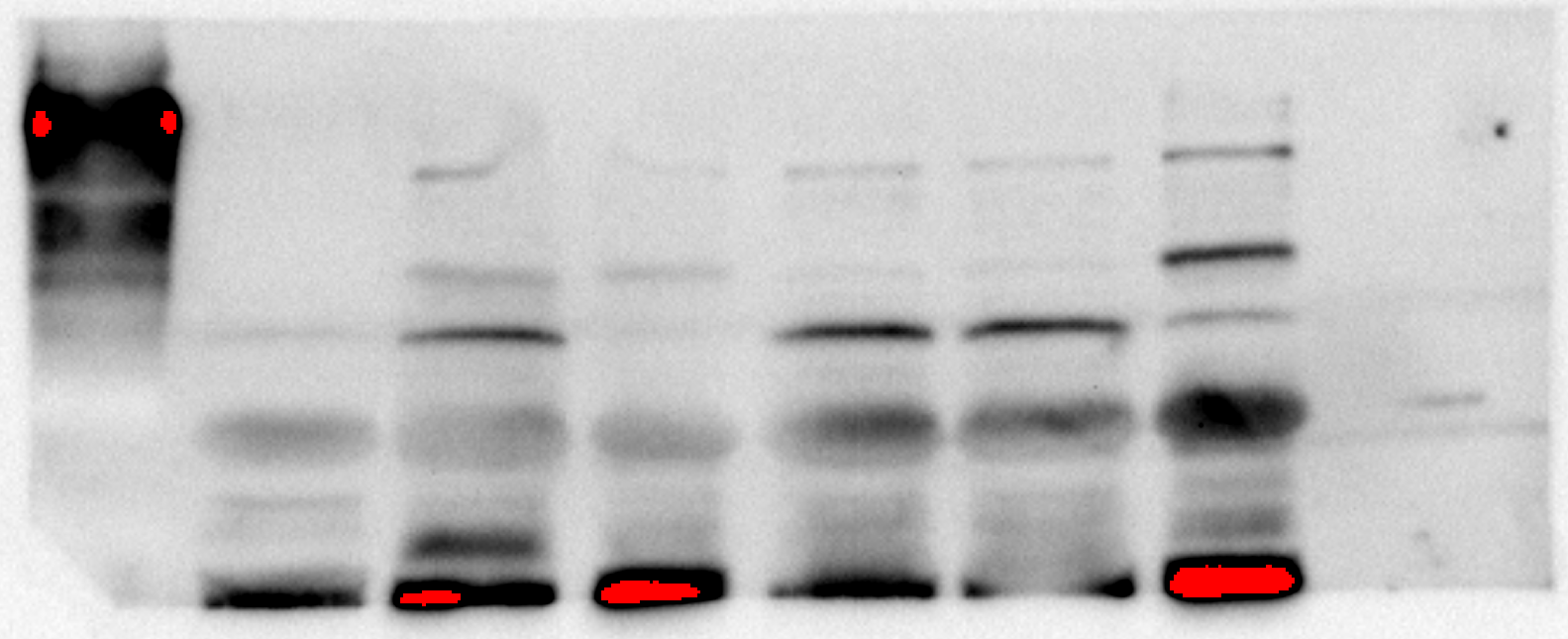

Supplement: Figure 4—source data 3. [file elife-90525-fig4-data3.zip › Figure 4-Source Data 3 [full raw unedited blot (bead bound-Atf1) of Figure 4A].tif]

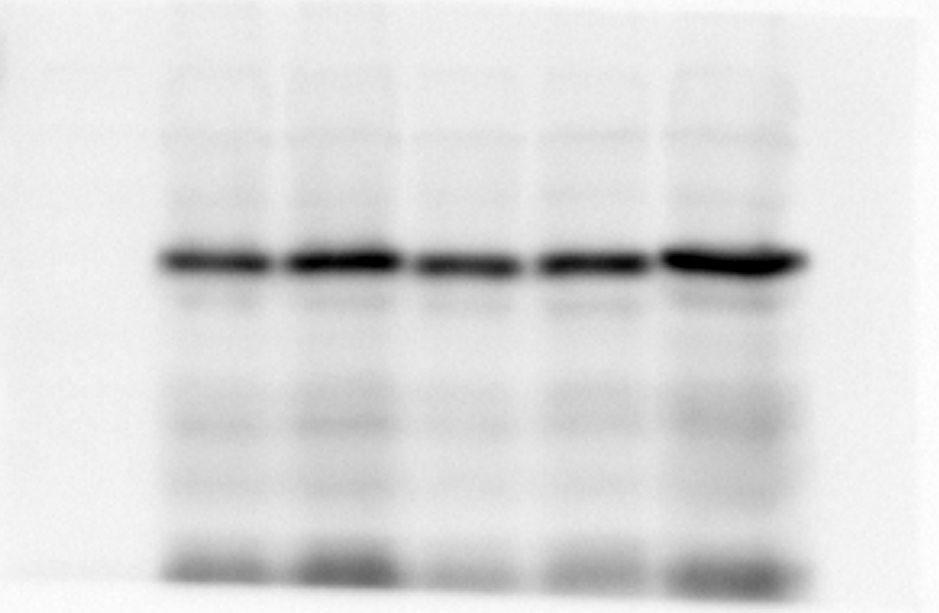

Supplement: Figure 4—source data 4. [file elife-90525-fig4-data4.zip › Figure 4-Source Data 4 [full raw unedited blot (WCE-Atf1) of Figure 4A].tif]

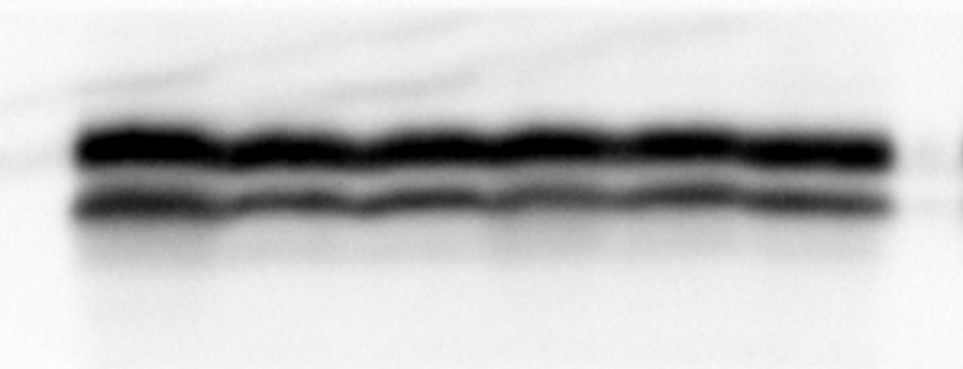

Supplement: Figure 4—source data 5. [file elife-90525-fig4-data5.zip › Figure 4-Source Data 5 [full raw unedited blot (WCE-Cdc2) of Figure 4A].tif]

Sun et al., Figure 4A.

A

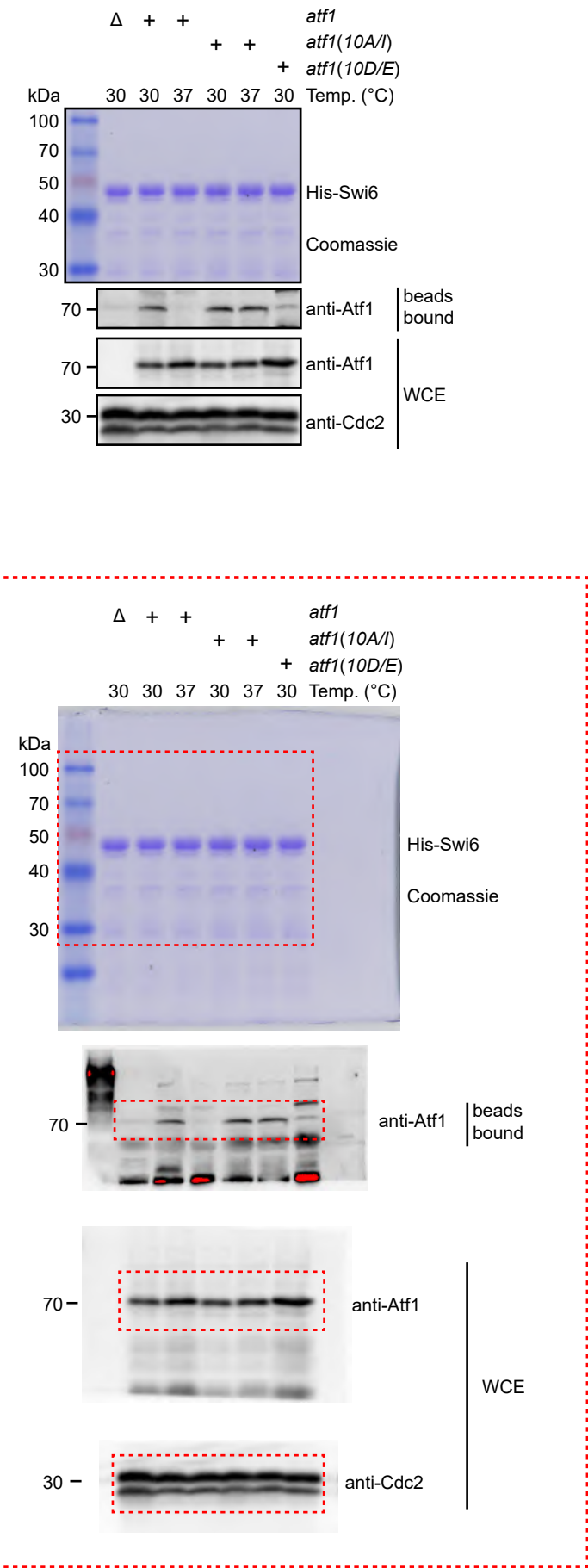

Supplement: Figure 4—source data 6. [file elife-90525-fig4-data6.pdf]

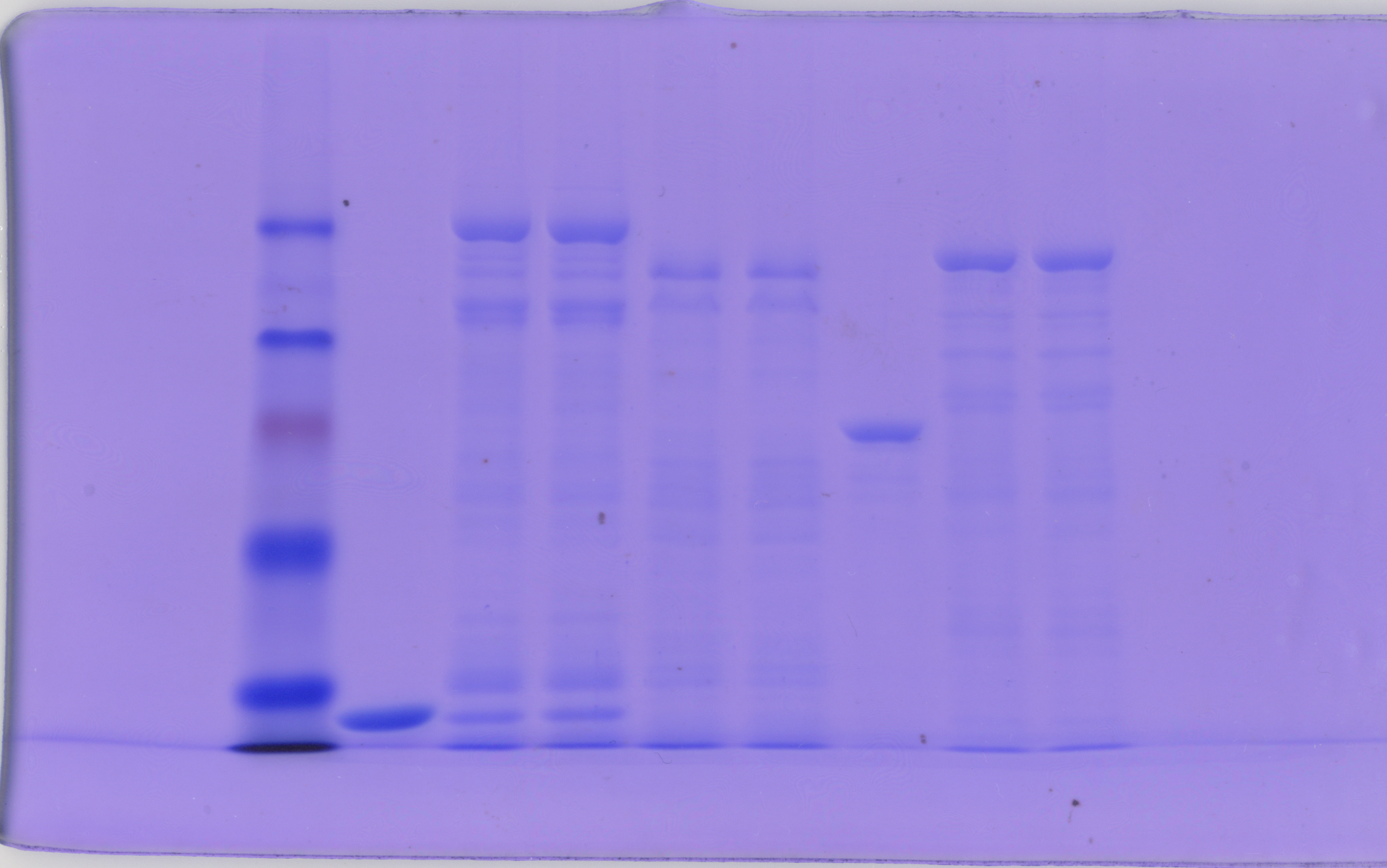

Supplement: Figure 4—figure supplement 1—source data 2. [file elife-90525-fig4-figsupp1-data2.zip › Figure 4-figure supplement 1-Source Data 2 [full raw unedited Coomassie gel for Figure A].tif]

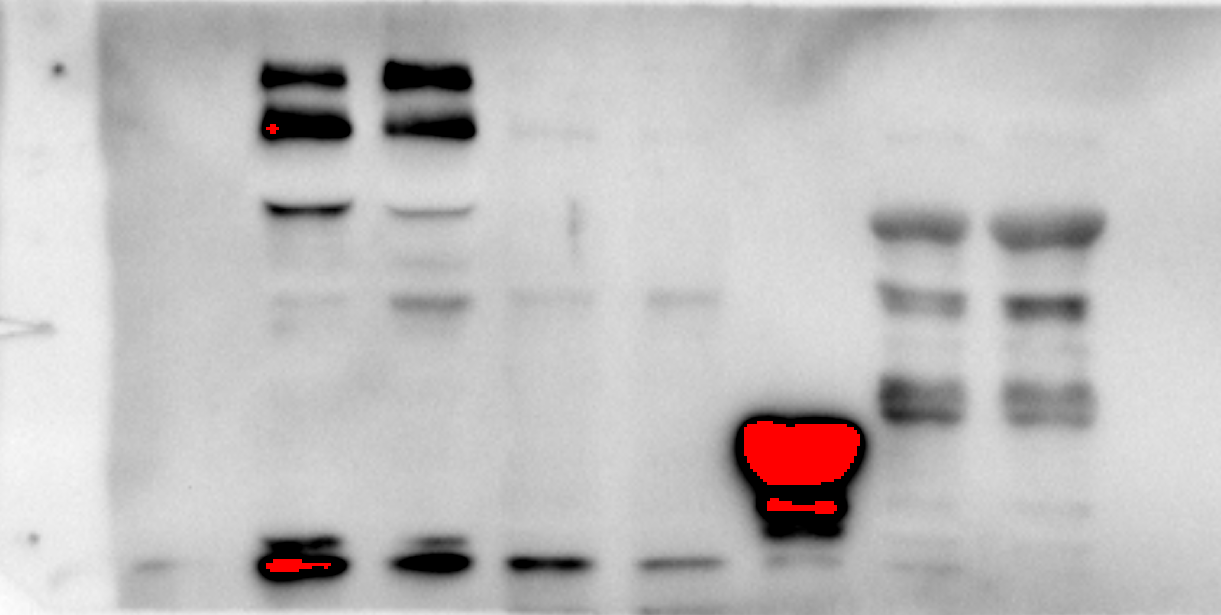

Supplement: Figure 4—figure supplement 1—source data 3. [file elife-90525-fig4-figsupp1-data3.zip › Figure 4-figure supplement 1-Source Data 3 [full raw unedited blot (bead bound-Atf1) for Figure A].tif]

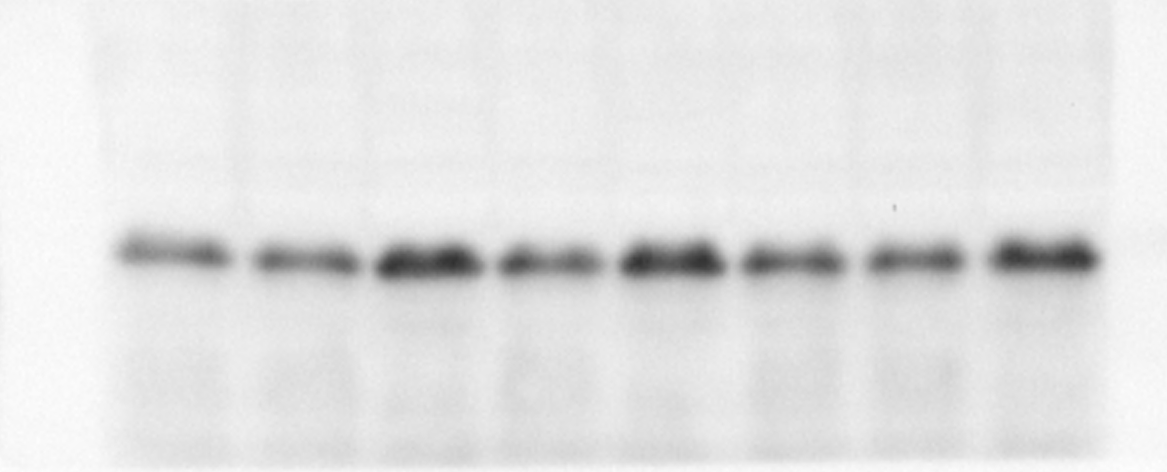

Supplement: Figure 4—figure supplement 1—source data 4. [file elife-90525-fig4-figsupp1-data4.zip › Figure 4-figure supplement 1-Source Data 4 [full raw unedited blot (WCE-Atf1) for Figure A].tif]

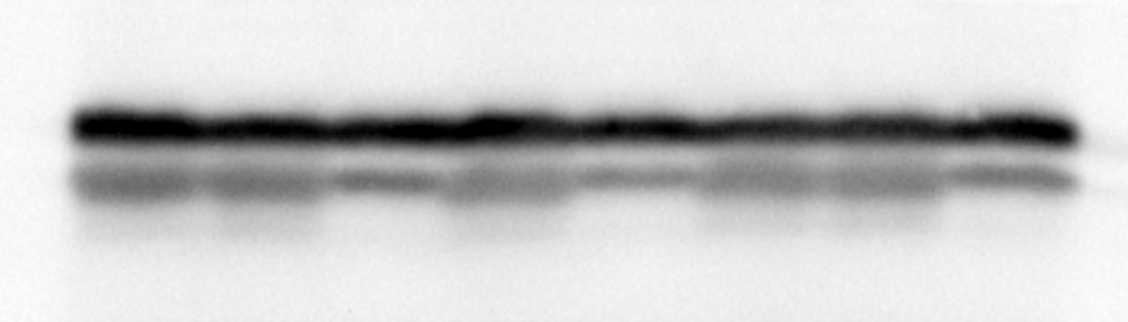

Supplement: Figure 4—figure supplement 1—source data 5. [file elife-90525-fig4-figsupp1-data5.zip › Figure 4-figure supplement 1-Source Data 5 [full raw unedited blot (WCE-Cdc2) for Figure A].tif]

Figure 4-figure supplement 1A.

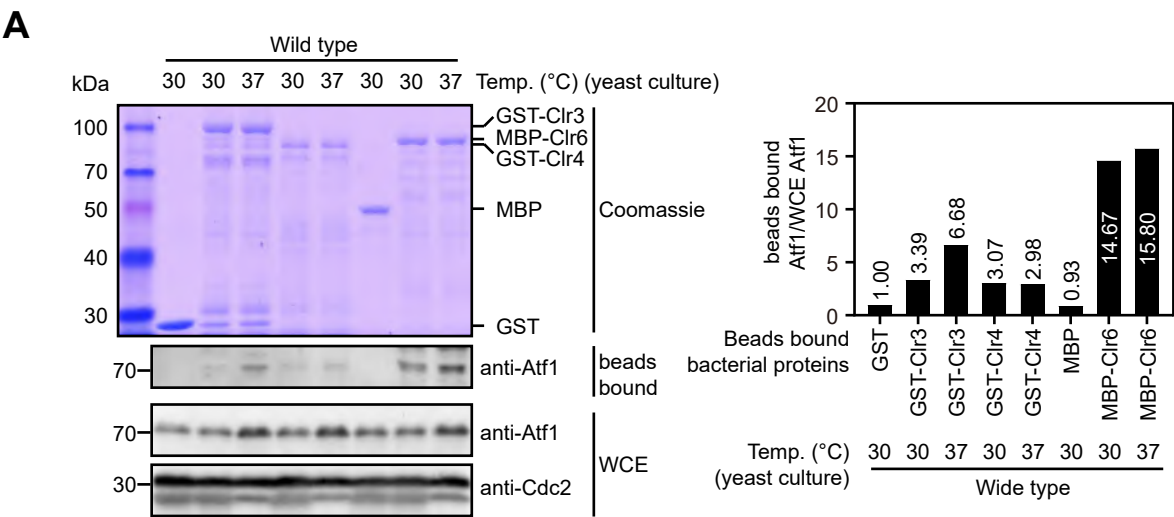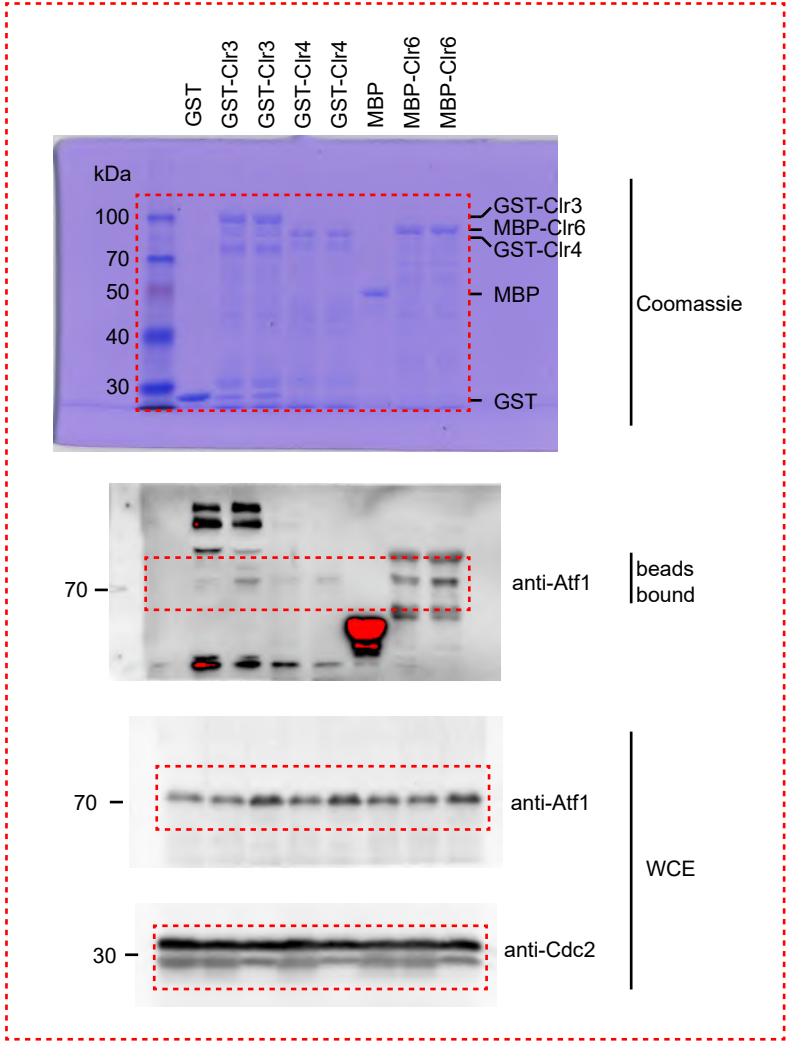

Supplement: Figure 4—figure supplement 1—source data 6. [file elife-90525-fig4-figsupp1-data6.pdf]

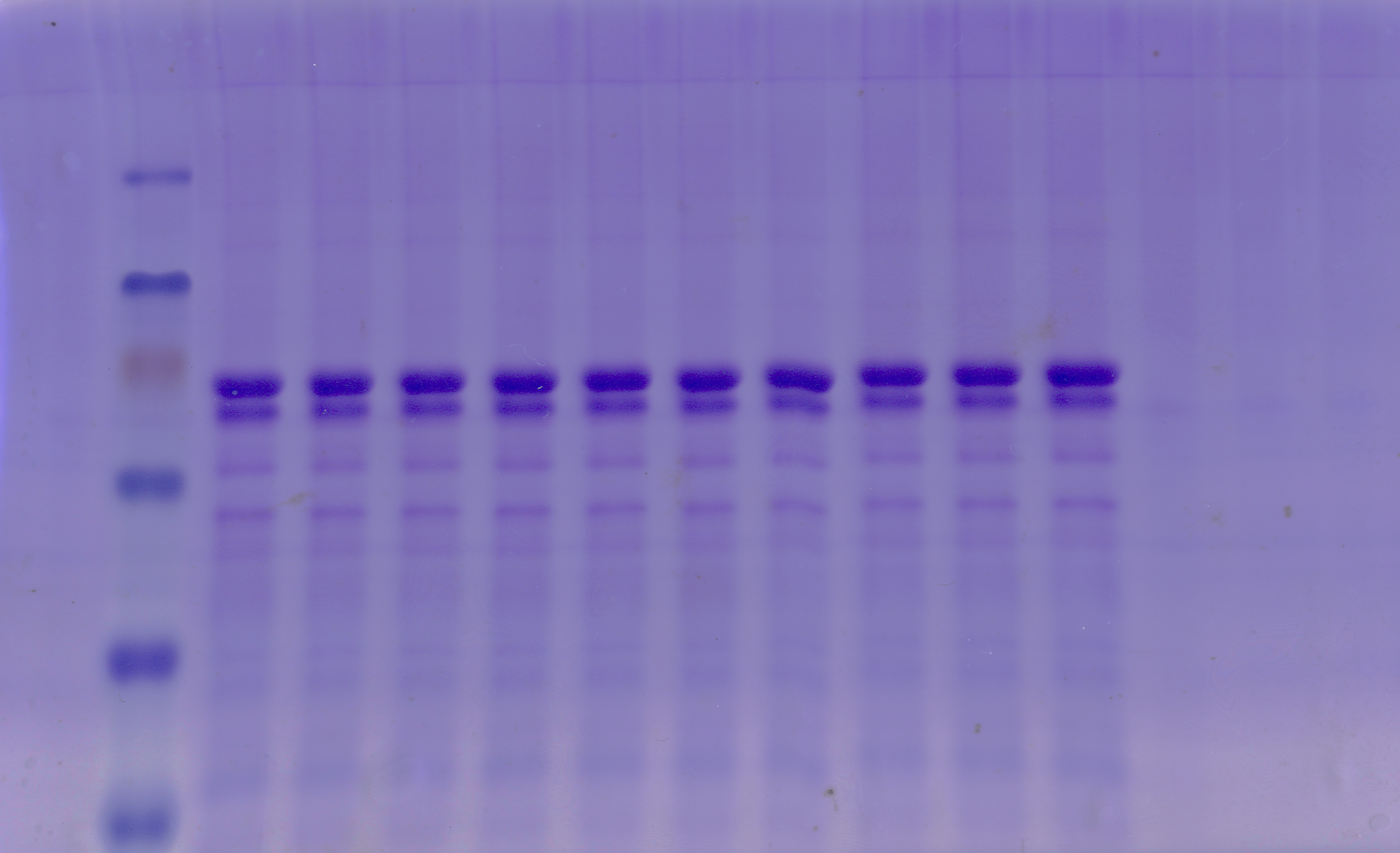

Supplement: Figure 4—figure supplement 2—source data 1. [file elife-90525-fig4-figsupp2-data1.zip › Figure 4-figure supplement 2-Source Data 1 [full raw unedited gel (Coomassie) for Figure A].tif]

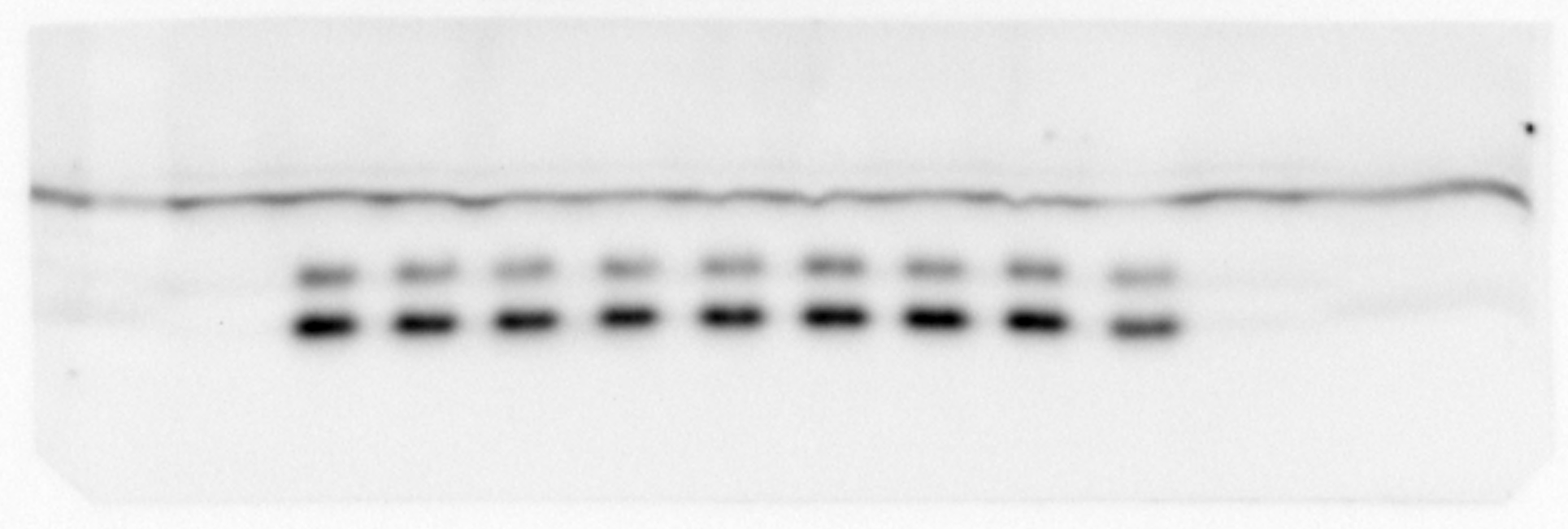

Supplement: Figure 4—figure supplement 2—source data 2. [file elife-90525-fig4-figsupp2-data2.zip › Figure 4-figure supplement 2-Source Data 2 [full raw unedited blot (bead bound-Pcr1x3Flag) for Figure A].tif]

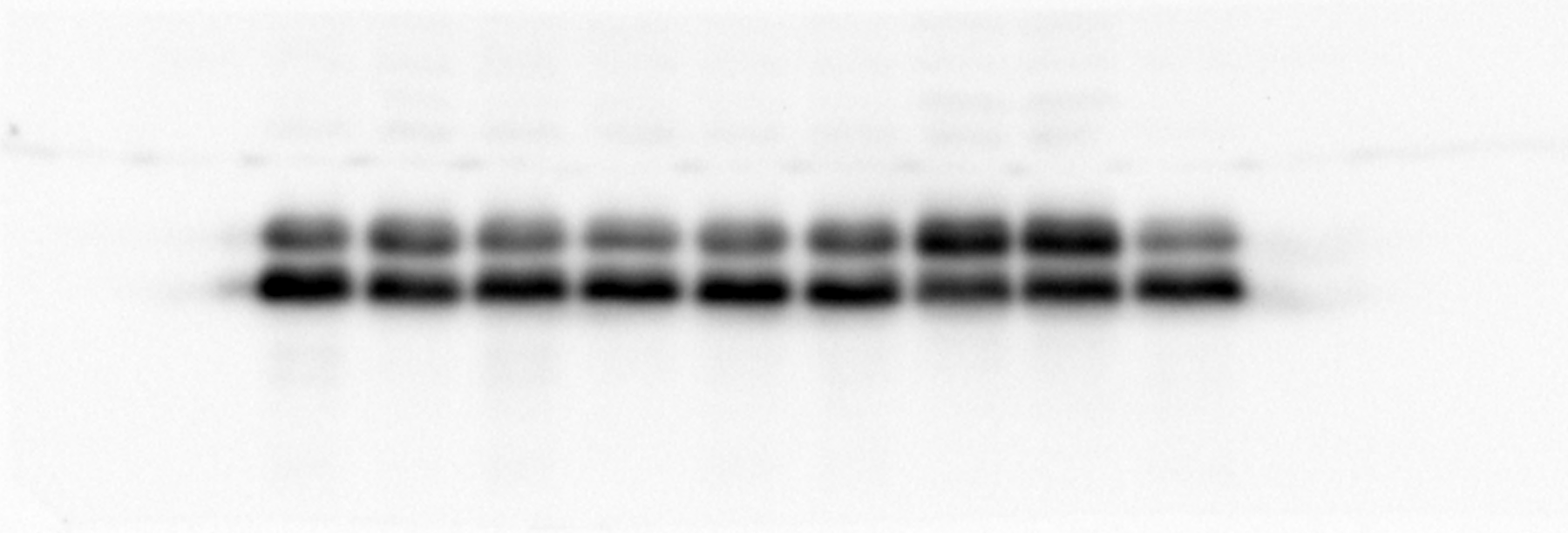

Supplement: Figure 4—figure supplement 2—source data 3. [file elife-90525-fig4-figsupp2-data3.zip › Figure 4-figure supplement 2-Source Data 3 [full raw unedited blot (WCE-Pcr1x3Flag) for Figure A].tif]

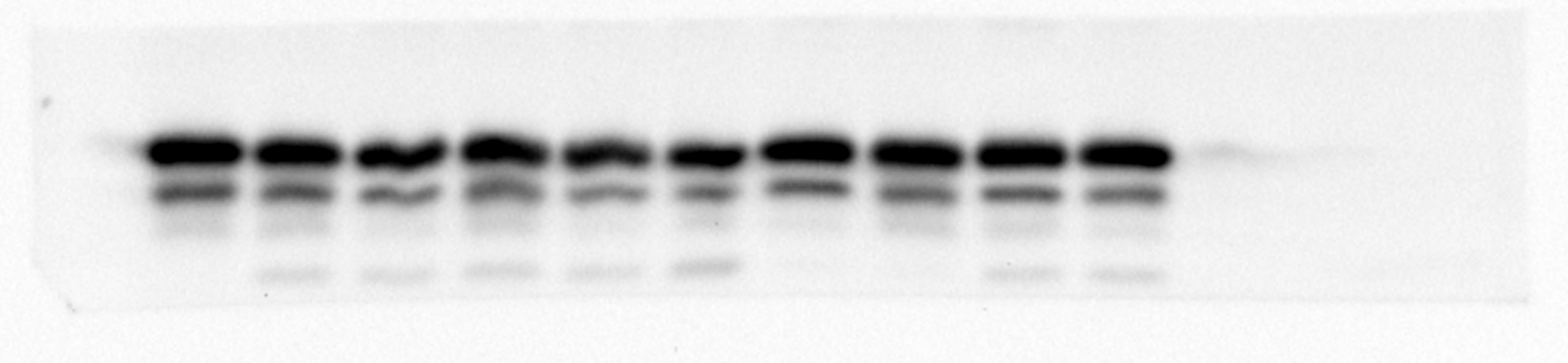

Supplement: Figure 4—figure supplement 2—source data 4. [file elife-90525-fig4-figsupp2-data4.zip › Figure 4-figure supplement 2-Source Data 4 [full raw unedited blot (WCE-Cdc2) for Figure A].tif]

Figure 4-figure supplement 2A

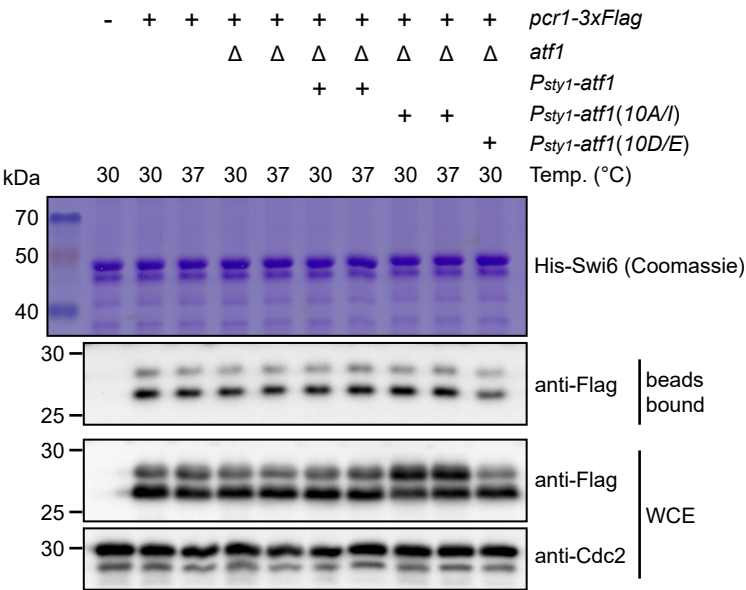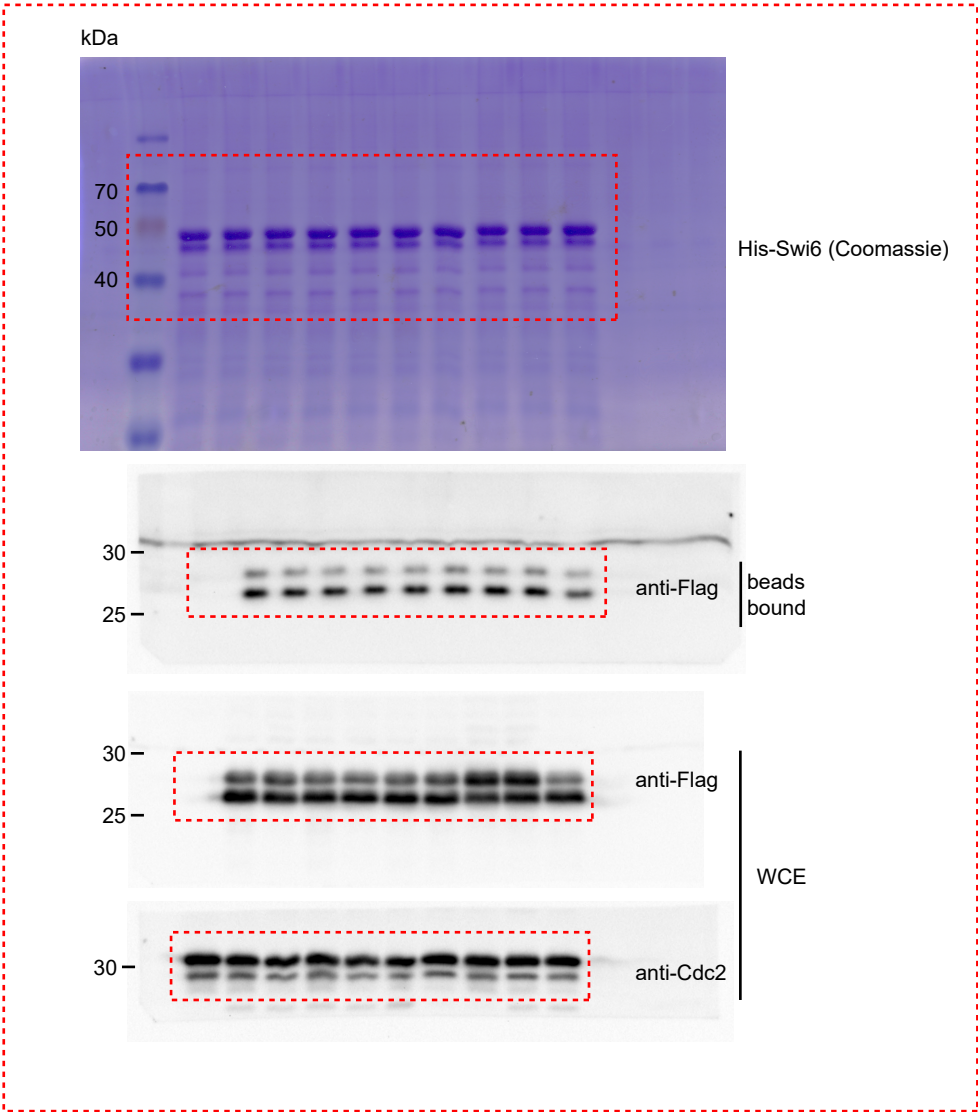

Supplement: Figure 4—figure supplement 2—source data 5. [file elife-90525-fig4-figsupp2-data5.pdf]

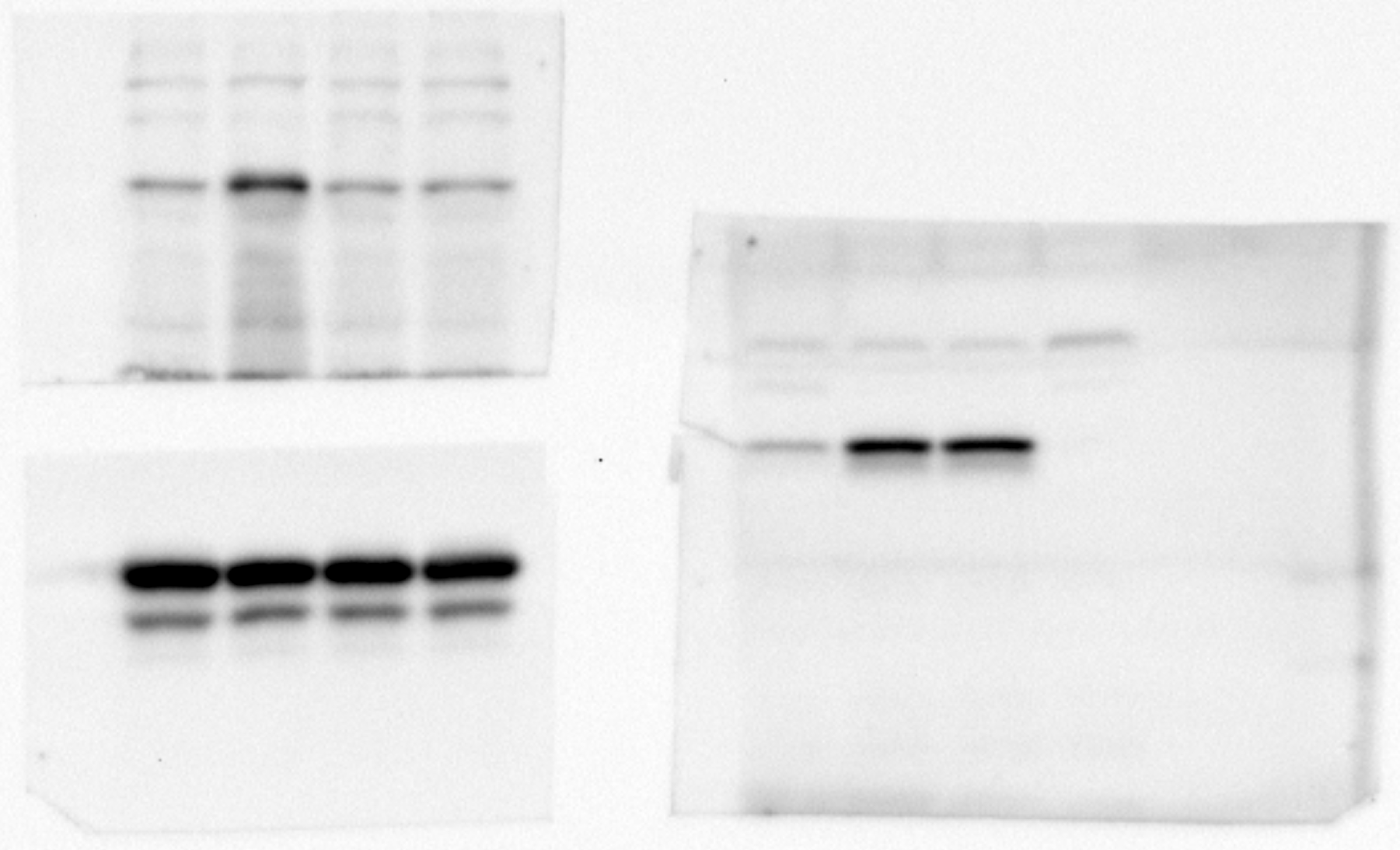

Supplement: Figure 5—source data 2. [file elife-90525-fig5-data2.zip › Figure 5-Source Data 2 [full raw unedited blots (Sty1-P, Atf1 and Cdc2) for Figure 5D].tif]

Figure 5D

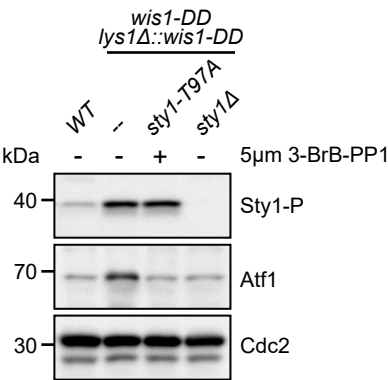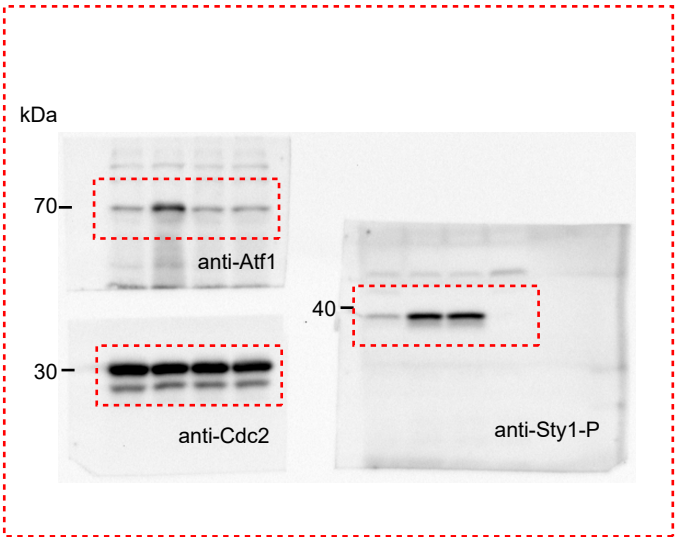

Supplement: Figure 5—source data 3. [file elife-90525-fig5-data3.pdf]

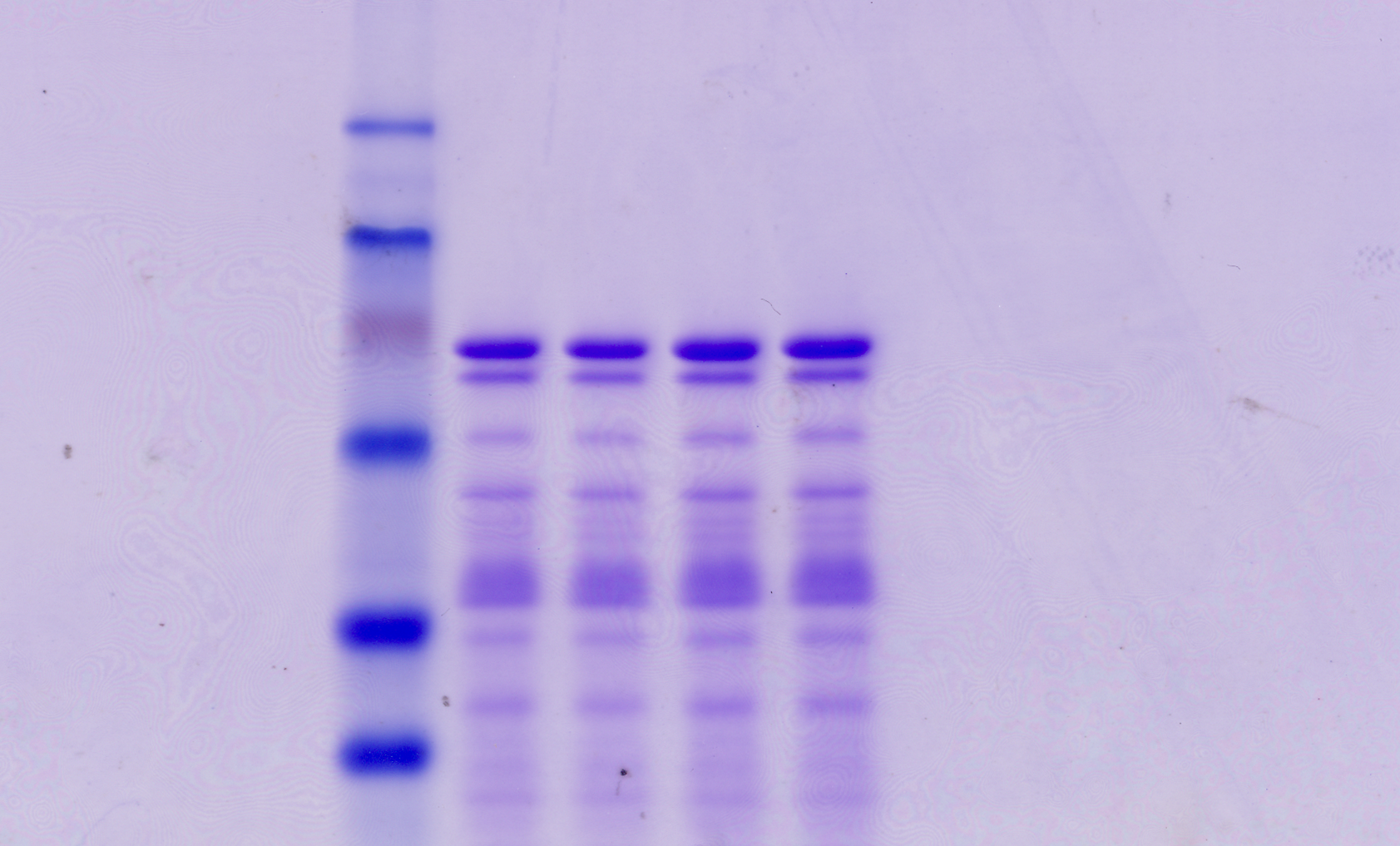

Supplement: Figure 5—source data 4. [file elife-90525-fig5-data4.zip › Figure 5-Source Data 4 [full raw unedited Coomassie gel for Figure 5E].tif]

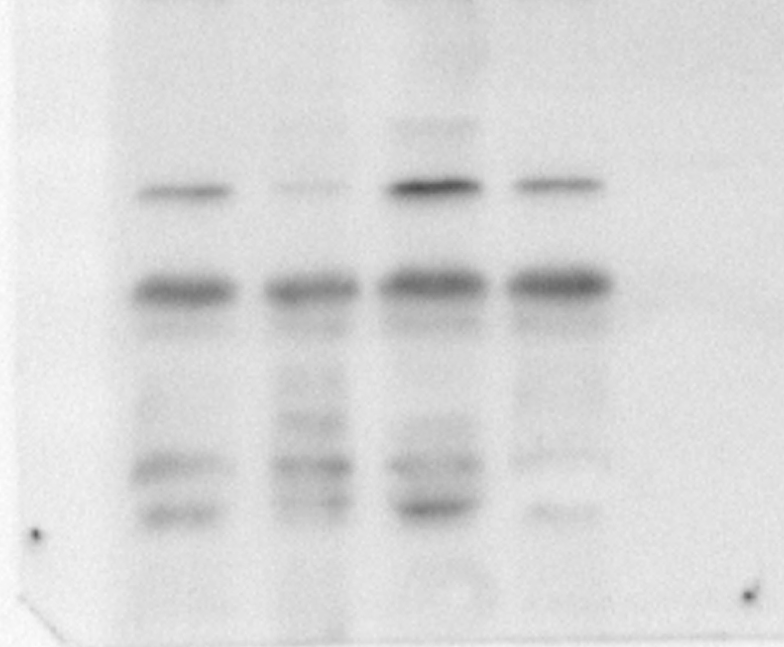

Supplement: Figure 5—source data 5. [file elife-90525-fig5-data5.zip › Figure 5-Source Data 5 [full raw unedited blot (bead bound-Atf1) for Figure 5E].tif]

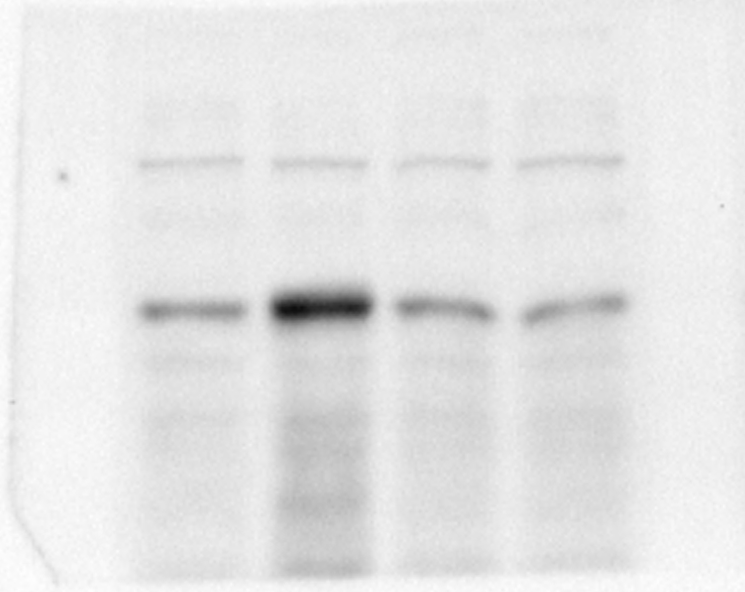

Supplement: Figure 5—source data 6. [file elife-90525-fig5-data6.zip › Figure 5-Source Data 6 [full raw unedited blot (WCE-Atf1) for Figure 5E].tif]

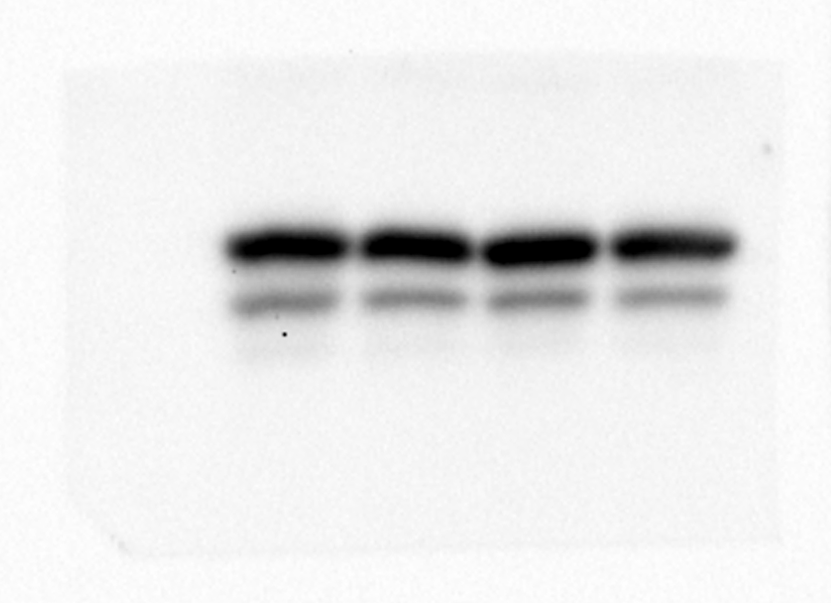

Supplement: Figure 5—source data 7. [file elife-90525-fig5-data7.zip › Figure 5-Source Data 7 [full raw unedited blot (WCE-Cdc2) for Figure 5E].tif]

Figure 5E

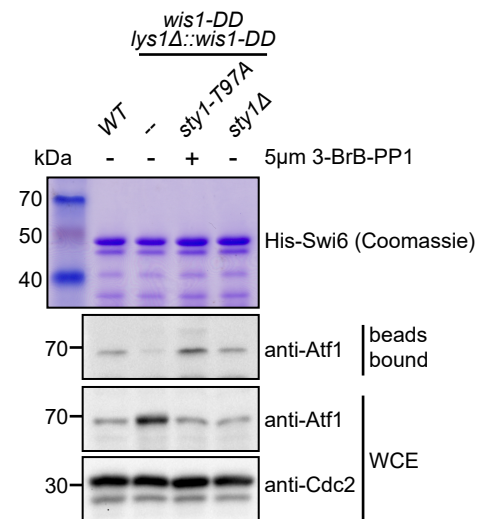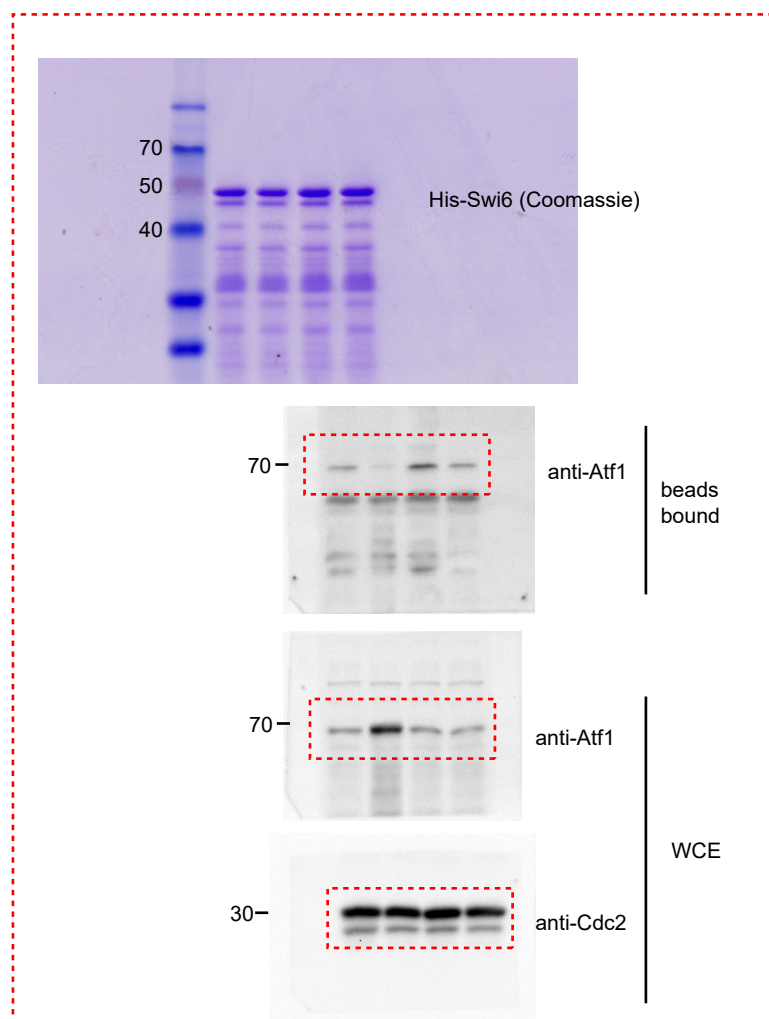

Supplement: Figure 5—source data 8. [file elife-90525-fig5-data8.pdf]

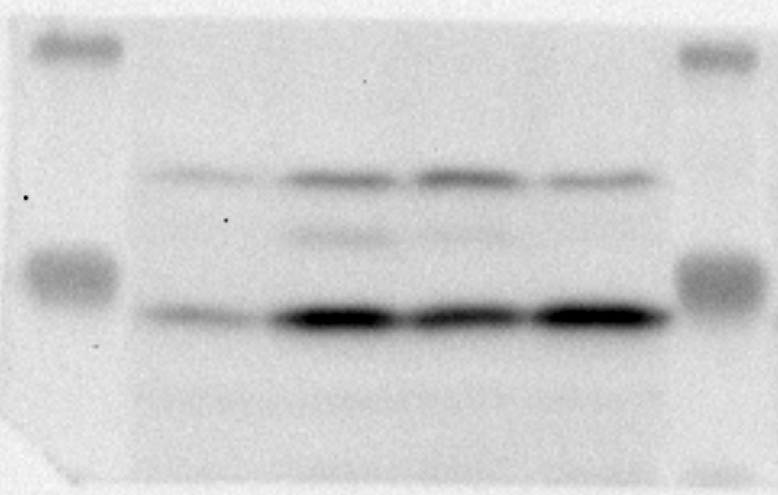

Supplement: Figure 5—figure supplement 1—source data 2. [file elife-90525-fig5-figsupp1-data2.zip › Figure 5-figure supplement 1-Source Data 2 [full raw unedited blot (Sty1-P) for Figure D].tif]

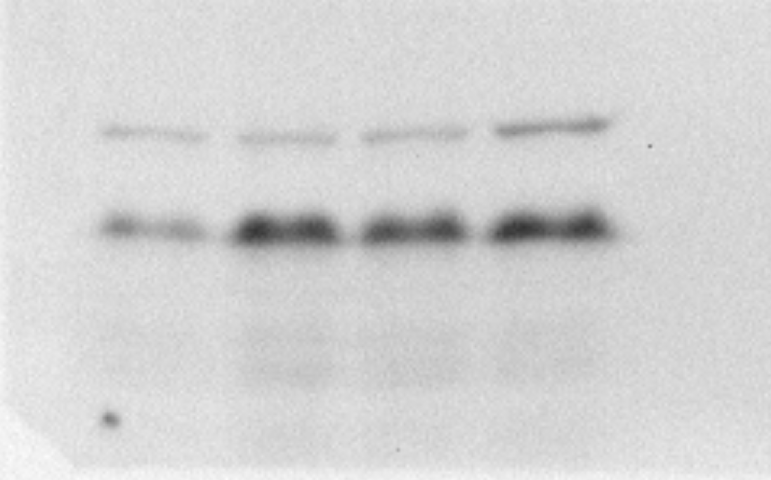

Supplement: Figure 5—figure supplement 1—source data 3. [file elife-90525-fig5-figsupp1-data3.zip › Figure 5-figure supplement 1-Source Data 3 [full raw unedited blot (Atf1) for Figure D].tif]

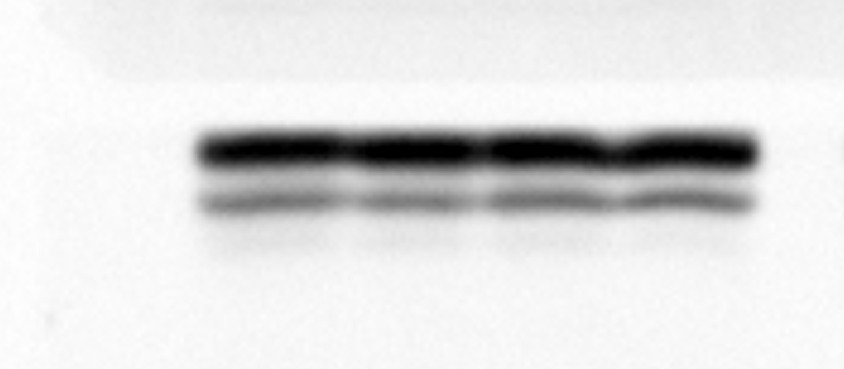

Supplement: Figure 5—figure supplement 1—source data 4. [file elife-90525-fig5-figsupp1-data4.zip › Figure 5-figure supplement 1-Source Data 4 [full raw unedited blot (Cdc2) for Figure D].tif]

Figure 5-figure supplement 1D.

**D**

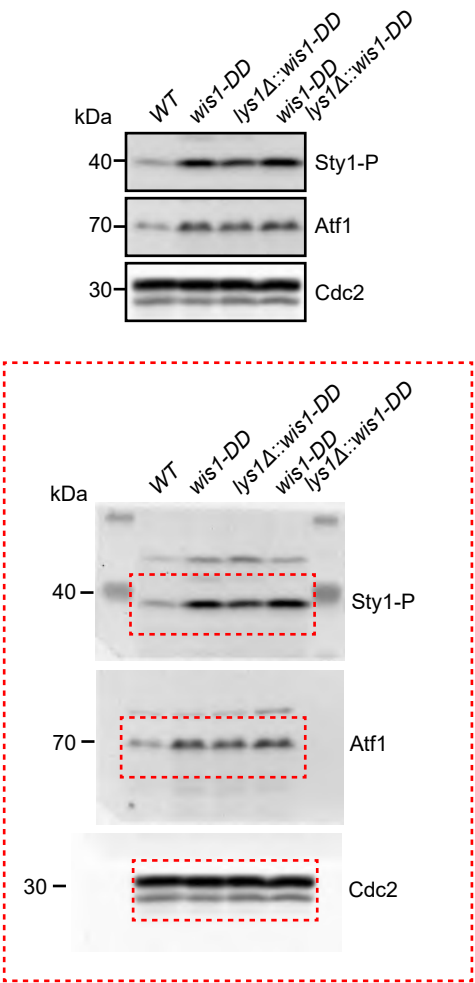

Supplement: Figure 5—figure supplement 1—source data 5. [file elife-90525-fig5-figsupp1-data5.pdf]

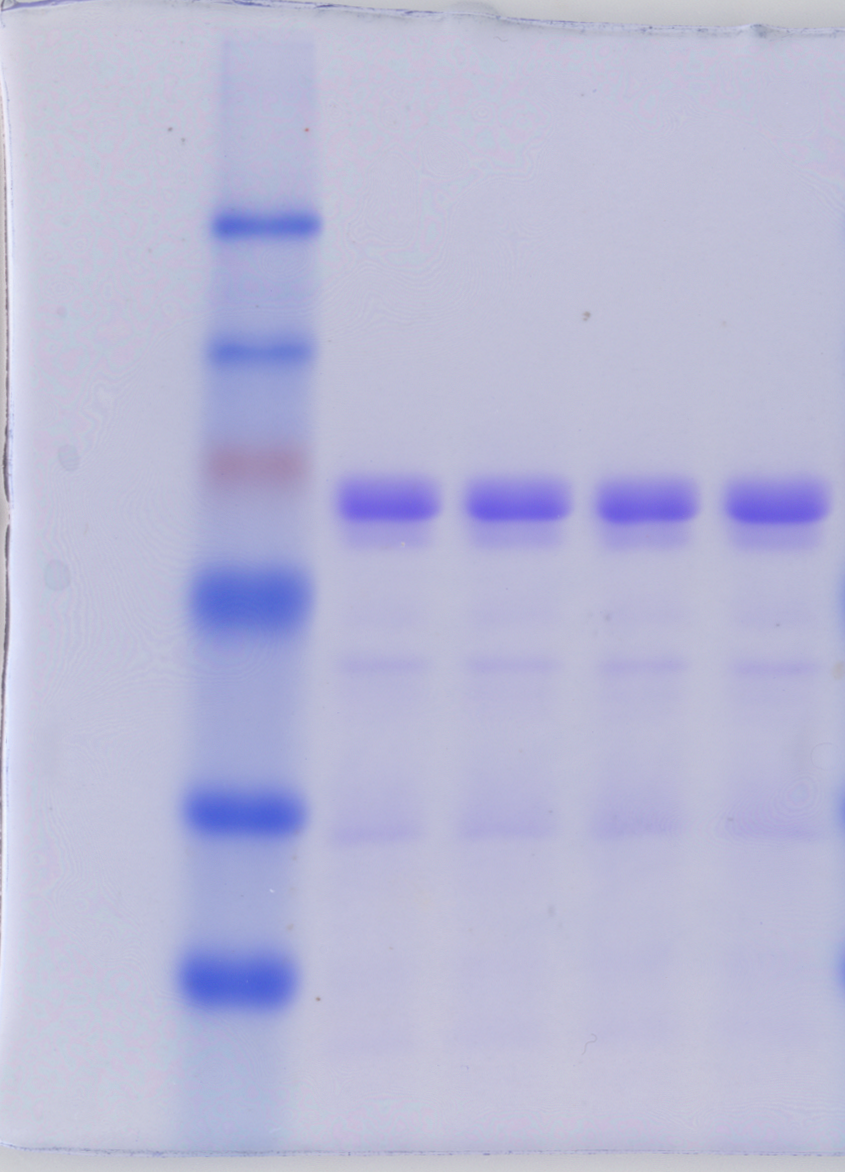

Supplement: Figure 5—figure supplement 1—source data 6. [file elife-90525-fig5-figsupp1-data6.zip › Figure 5-figure supplement 1-Source Data 6 [full raw unedited gel (Coomassie) for Figure E].tif]

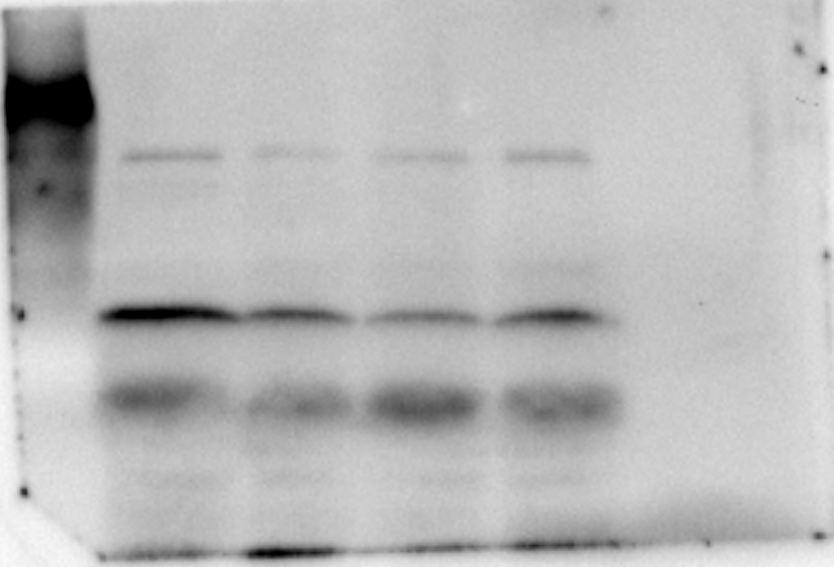

Supplement: Figure 5—figure supplement 1—source data 7. [file elife-90525-fig5-figsupp1-data7.zip › Figure 5-figure supplement 1-Source Data 7 [full raw unedited blot (beads bound-Atf1) for Figure E].tif]

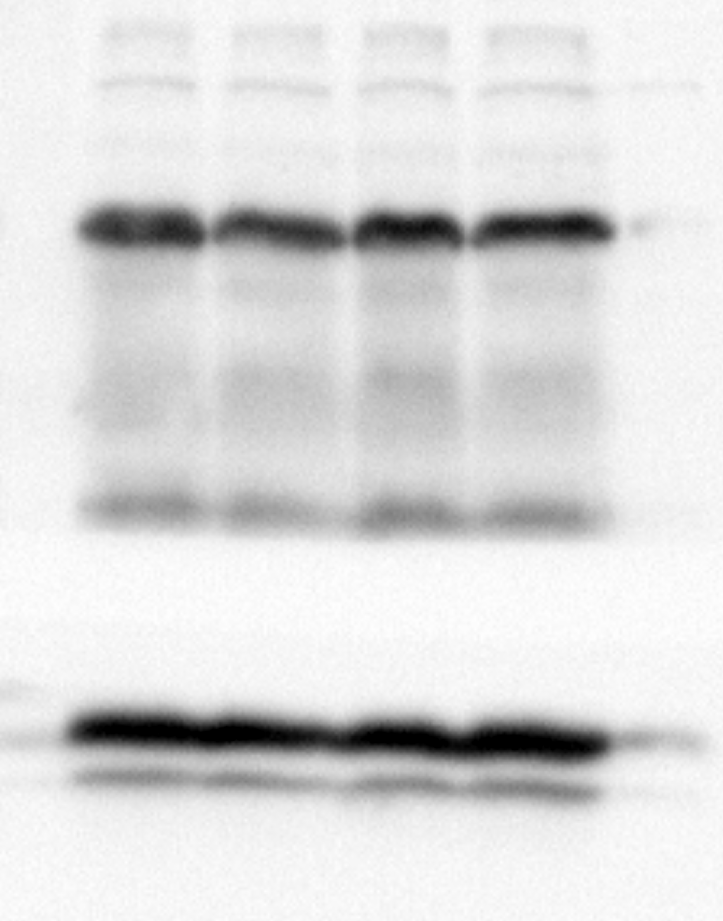

Supplement: Figure 5—figure supplement 1—source data 8. [file elife-90525-fig5-figsupp1-data8.zip › Figure 5-figure supplement 1-Source Data 8 [full raw unedited blot (WCE-Atf1 and Cdc2) for Figure E].tif]

Figure 5-figure supplement 1E.

E

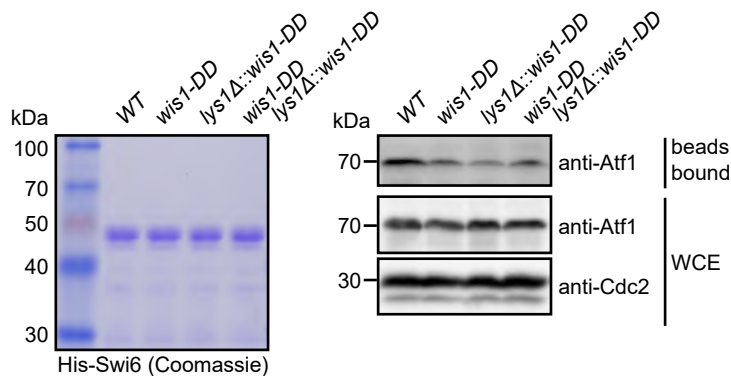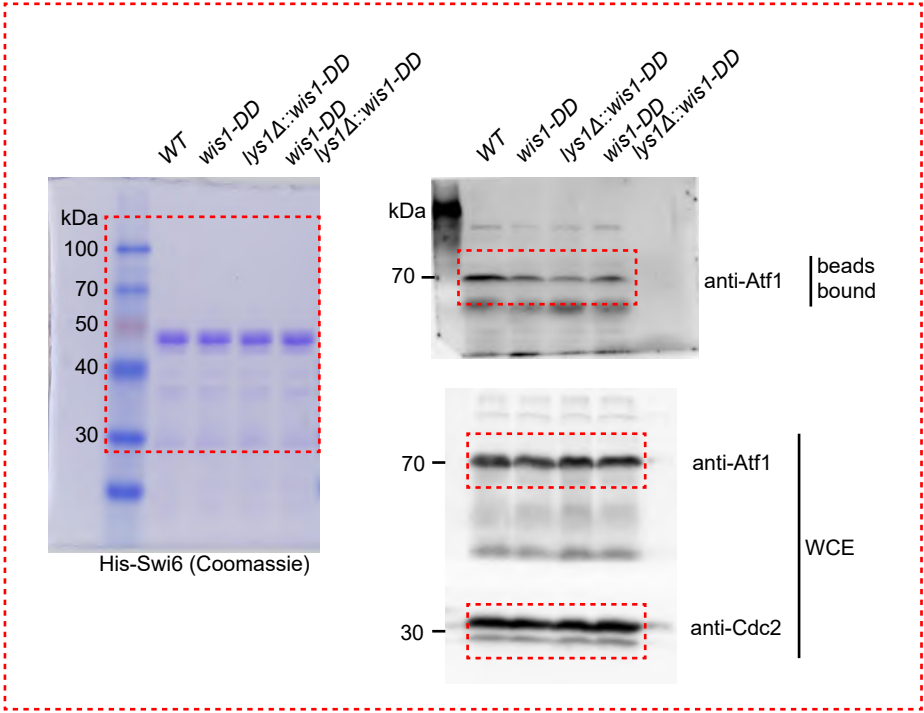

Supplement: Figure 5—figure supplement 1—source data 9. [file elife-90525-fig5-figsupp1-data9.pdf]
