# Supplementary material for: Heat stress-induced activation of MAPK pathway attenuates Atf1-dependent epigenetic inheritance of heterochromatin in fission yeast
Source: eLife. 2024 Jan 30;13:e90525. doi: 10.7554/eLife.90525 (PMC10863984; doi:10.7554/eLife.90525)
Supplement: Supplementary file 1. — (a) Yeast strains used in this study. (b) Primers used for RT-qPCR and qPCR. [file elife-90525-supp1.doc]

**Supplementary file 1a. Yeast strains used in this study.**

| Strain | Genotype | Source |
| --- | --- | --- |
| JY3 | *h^-^ ade6-216 leu1-32 ura4-D18* | Lab stock |
| JY4 | *h^+^ ade6-216 leu1-32 ura4-D18* | Lab stock |
| JY992 | *h^-^ leu1-32 ura4-D18 ade6-DN/N* | Lab stock |
| JY8499 | *h^+^ leu1-32 ura4-D18 ade6-DN/N* | Lab stock |
| JY972 | *h^90^ mat3M(Eco*RV*)::ade6^+^ leu1-32 ura4-D18 ade6-DN/N* | Lab stock |
| JY1543 | *h^-^ otr1R(Sph*I*)::ade6^+^ ade6-DN/N leu1-32 ura4-D18* | Lab stock |
| JY104 | *h^-^ ndc80-GFP::kan^R^ leu1-32 ura4-D18 ade6^+^* | Lab stock |
| JY115 | *h^+^ TM-ura4^+^ ade6-210 leu1-32 ura4-DS/E* | Lab stock |
| JY117 | *h^+^ imr1R (Nco*I*)::ura4^+^ ade6-210 leu1-32 ura4-DS/E* | Lab stock |
| JY118 | *h^+^ otr1R(Sph*I*)::ura4^+^ ade6-210 leu1-32 ura4-DS/E* | Lab stock |
| JY1092 | *mat1-Msmt-0 mat2P(Xba*Ⅰ*)::ura4^+^ ade6-216 ura4-D18* | Lab stock |
| JY390 | *h^90^ mat3M(Eco*RV*)::ade6^+^ leu1-32 ura4-D18 ade6-DN/N* | Lab stock |
| JY8956 | *h^90^ tel1L::his3^+^ tel2L::ura4^+^ leu1-32 his3-D1 ade6-210 ura4-D18* | Thomas S van Emden et al., 2018 |
| JY7240 | *h^-^ rDNA::ura4^+^ leu1-32 ura4-D18 ade6-210* | Lab stock |
| JY9755 | *h^+^ imr1R(Nco*I*)::gfp^+^::nat^R^ leu1-32 ura4-D18 ade6-216 [gfp^+^ driven by ura4^+^ promoter]* | Claudia Keller et al., 2012 |
| JY9765 | *h^-^ imr1R(Nco*I*)::gfp^+^::nat^R^ swi6∆::ura4^+^ leu1-32 ura4-D18 [gfp^+^ driven by ura4^+^ promoter]* | Claudia Keller et al., 2012 |
| JY9769 | *h^-^ imr1R(Nco*I*)::gfp^+^::natMX dcr1∆::kan^R^ leu1-32 ura4-D18 [gfp^+^ driven by ura4^+^ promoter]* | Claudia Keller et al., 2012 |
| JY9763 | *h^90^ mat3M(Eco*RV*)::gfp^+^::nat^R^ swi6∆::ura4^+^ leu1-32* | Claudia Keller et al., 2012 |
| JY9773 | *h^90^ mat3M(Eco*RV*)::gfp^+^::nat^R^ leu1-32* | Claudia Keller et al., 2012 |
| JY9786 | *h^90^ mat3M(Eco*RV*)::gfp^+^::nat^R^ dcr1∆::kan^R^ leu1-32* | Claudia Keller et al., 2012 |
| JY9142 | *h^90^ mat3M(Eco*RV*)::ade6^+^ dcr1∆::kan^R^ ade6-DN/N*  *leu1-32* | This study |
| JY9092 | *h^90^ mat3M(Eco*RV*)::ade6^+^ clr4∆::kan^R^ ade6-DN/N*  *leu1-32* | This study |
| JY9215 | *h^90^ mat3M(Eco*RV*)::ade6^+^ atf1Δ::nat^R^ ade6-DN/N* | This study |
| JY9216 | *h^90^ mat3M(Eco*RV*)::ade6^+^ atf1Δ::nat^R^ ade6-DN/N*  *P_sty1_::HA-atf1::leu1^+^* | This study |
| JY8478 | *h^90^ mat3M(Eco*RV*)::ade6^+^ atf1Δ::nat^R^ ade6-DN/N*  *P_sty1_::HA-atf1(10M)::leu1^+^* | This study |
| JY8480 | *h^90^ mat3M(Eco*RV*)::ade6^+^ atf1Δ::nat^R^ ade6-DN/N*  *P_sty1_::HA-atf1(10D)::leu1^+^* | This study |
| JY9283 | *h^90^ mat3M(EcoRV)::ade6^+^ 3xFlag-clr4 ade6-DN/N* | Lab stock |
| JY9285 | *h^90^ mat3M(EcoRV)::ade6^+^ clr3-myc::kan^R^ ade6-DN/N ura4-D18 leu1-32* | This study |
| JY9681 | *h^90^ mat3M(EcoRV)::ade6^+^ epe1-3HA::kan^R^ ade6-DN/N ura4-D18 leu1-32* | This study |
| JY11038 | *h^90^ mat3M(EcoRV)::ade6^+^ clr6-13myc::hyg^R^ ade6-DN/N ura4-D18 leu1-32* | This study |
| JY9277 | *h^90^ mat3M(Eco*RV*)::4xtetO-ade6^+^ ade6-DN/N leu1-32 ura4-D18* | This study |
| JY10680 | *h^90^ mat3M(Eco*RV*)::4xtetO-ade6^+^ ade6-DN/N leu1-32 ura4-D18 lys1Δ::P_adh81_-swi6^ΔCD^::hyg^R^* | This study |
| JY10681 | *h^90^ mat3M(Eco*RV*)::4xtetO-ade6^+^ ade6-DN/N leu1-32 ura4-D18 lys1Δ::P_adh81_-TetR^off^-2xFlag::hyg^R^* | This study |
| JY10644 | *h^90^ mat3M(Eco*RV*)::4xtetO-ade6^+^ ade6-DN/N leu1-32 ura4-D18 lys1Δ::P_adh81_-TetR^off^-2xFlag-swi6^ΔCD^::hyg^R^* | This study |
| JY8771 | *mat1-Msmt-0 KΔ::ade6^+/off^ ade6-DN/N ura4-D18* | This study |
| JY8772 | *mat1-Msmt-0 KΔ::ade6^+/off^ wis1-DD::ura4^+^ ade6-DN/N ura4-D18* | This study |
| JY10747 | *mat1-Msmt-0 KΔ::ade6^+/off^ ade6-DN/N ura4-D18 lys1Δ::P_ahd11_-HA-wis1-DD::hyg^R^* | This study |
| JY10748 | *mat1-Msmt-0 KΔ::ade6^+/off^ wis1-DD::ura4^+^ ade6-DN/N ura4-D18 lys1Δ::P_ahd11_-HA-wis1-DD::hyg^R^* | This study |
| JY9574 | *h^90^ mat3M(Eco*RV*)::ade6^+^ epe1∆::hyg^R^ ade6-DN/N*  *ura4-D18 leu1-32* | This study |
| JY9550 | *h^90^ mat3M(Eco*RV*)::ade6^+^ mst2∆::hyg^R^ ade6-DN/N*  *ura4-D18 leu1-32* | This study |
| JY10478 | *h^90^ mat3M(Eco*RV*)::ade6^+^ leo1∆::hyg^R^ ade6-DN/N*  *ura4-D18 leu1-32* | This study |
| JY10313 | *h^90^ mat3M(Eco*RV*)::ade6^+^ taz1∆::kan^R^ ade6-DN/N*  *leu1-32 ura4-D18* | This study |
| JY10314 | *h^90^ mat3M(Eco*RV*)::ade6^+^ rap1∆::kan^R^ ade6-DN/N*  *leu1-32 ura4-D18* | This study |
| JY10349 | *h^90^ mat3M(Eco*RV*)::ade6^+^ poz1∆::hyg^R^ ade6-DN/N*  *leu1-32 ura4-D18* | This study |
| JY10372 | *h^90^ mat3M(Eco*RV*)::ade6^+^ ccq1∆::hyg^R^ ade6-DN/N*  *leu1-32 ura4-D18* | This study |
| JY10242 | *h^90^ mat3M(Eco*RV*)::ade6^+^ atf1(10A/I) ade6-DN/N leu1-32 ura4-D18* | This study |
| JY10469 | *h^90^ mat3M(Eco*RV*)::ade6^+^ atf1(10D/E) ade6-DN/N leu1-32 ura4-D18* | This study |
| JY11211 | *h^90^ mat3M(Eco*RV*)::ade6^+^ atf1Δ::nat^R^ ade6-DN/N pcr1-3Flag::kan^R^* | This study |
| JY11212 | *h^90^ mat3M(Eco*RV*)::ade6^+^ atf1Δ::nat^R^ ade6-DN/N P_sty1_::HA-atf1::leu1^+^ pcr1-3Flag::kan^R^* | This study |
| JY11213 | *h^90^ mat3M(Eco*RV*)::ade6^+^ atf1Δ::natR ade6-DN/N P_sty1_::HA-atf1(10A/I)::leu1^+^ pcr1-3Flag::kan^R^* | This study |
| JY11214 | *h^90^ mat3M(Eco*RV*)::ade6+ atf1Δ::nat^R^ ade6-DN/N P_sty1_::HA-atf1(10D/E)::leu1^+^ pcr1-3Flag::kan^R^* | This study |
| JY11215 | *h^90^ mat3M(Eco*RV*)::ade6^+^ ade6-DN/N pcr1-3Flag::kan^R^* | This study |
| JY11238 | *mat1-Msmt-0 KΔ::ade6^+/off^ ade6-DN/N ura4-D18 sty1-T97A::nat^R^* | This study |
| JY11239 | *mat1-Msmt-0 KΔ::ade6^+/off^ wis1-DD::ura4^+^ ade6-DN/N ura4-D18 sty1-T97A::nat^R^* | This study |
| JY11240 | mat1-Msmt-0 KΔ::ade6^+/off^ ade6-DN/N ura4-D18  lys1Δ::P_ahd11_-HA-wis1-DD::hyg^R^ *sty1-T97A::nat^R^* | This study |
| JY11241 | mat1-Msmt-0 KΔ::ade6^+/off^ *wis1-DD::ura4^+^* ade6-DN/N ura4-D18  lys1Δ::P_ahd11_-HA-wis1-DD::hyg^R^ *sty1-T97A::nat^R^* | This study |
| JY11279 | *mat1-Msmt-0 KΔ::ade6^+/off^ wis1-DD::ura4^+^ sty1∆::nat^R^*  *ade6-DN/N ura4-D18* | This study |
| JY11280 | *mat1-Msmt-0 KΔ::ade6^+/off^ sty1∆::nat^R^*  *lys1Δ::P_adh11_-6xHA-wis1-DD::hyg^R^ ade6-DN/N ura4-D18* | This study |
| JY11281 | *mat1-Msmt-0 KΔ::ade6^+/off^ sty1∆::nat^R^ ade6-DN/N ura4-D18* | This study |
| JY11282 | *mat1-Msmt-0 KΔ::ade6^+/off^ wis1-DD::ura4^+^ sty1∆::nat^R^*  *lys1Δ::P_adh11_-6xHA-wis1-DD::hyg^R^ ade6-DN/N ura4-D18* | This study |
| JY11246 | *h^90^ mat3M(Eco*RV*)::ade6^+^ atf1Δ::nat^R^ ade6-DN/N*  *leu1-32::P_sty1_-HA-atf1(11A/I)::leu1^+^ ura4-D18* | This study |
| JY11247 | *h^90^ mat3M(Eco*RV*)::ade6^+^ atf1Δ::nat^R^ ade6-DN/N*  *leu1-32::P_sty1_-HA-atf1(11D/E)::leu1^+^ ura4-D18* | This study |
| JY11312 | *h^90^ mat3M(Eco*RV*)::ade6^+^ atf1Δ::nat^R^ ade6-DN/N*  *leu1-32::P_sty1_-HA-atf1(S438A)::leu1^+^ ura4-D18* | This study |
| JY11313 | *h^90^ mat3M(Eco*RV*)::ade6^+^ atf1Δ::nat^R^ ade6-DN/N*  *leu1-32::P_sty1_-HA-atf1(S438D)::leu1^+^ ura4-D18* | This study |
| JY11351 | *h^90^ mat3M(Eco*RV*)::ade6^+^ atf1Δ::nat^R^ ade6-DN/N*  *leu1-32::P_sty1_-HA-atf1(4A/I)::leu1^+^ ura4-D18* | This study |
| JY11352 | *h^90^ mat3M(Eco*RV*)::ade6^+^ atf1Δ::nat^R^ ade6-DN/N*  *leu1-32::P_sty1_-HA-atf1(4D/E)::leu1^+^ ura4-D18* | This study |
| JY11353 | *h^90^ mat3M(Eco*RV*)::ade6^+^ atf1Δ::nat^R^ ade6-DN/N*  *leu1-32::P_sty1_-HA-atf1(6A/I)::leu1^+^ ura4-D18* | This study |
| JY11354 | *h^90^ mat3M(Eco*RV*)::ade6^+^ atf1Δ::nat^R^ ade6-DN/N*  *leu1-32::P_sty1_-HA-atf1(6D/E)::leu1^+^ ura4-D18* | This study |
|  | | |

**Supplementary file 1b. Primers used for RT-qPCR and qPCR.**

| Name | Sequence (5’🡪3’) |
| --- | --- |
| cen_dg-F | GTGCCTTTCCAAGTTAGGGG |
| cen_dg-R | CACAGAAGCATCCACTGGGC |
| mat2-Pc-RT-F | CCCTGCTTATATGTAGTTTATAATTGTTGTGTCC |
| mat2-Pc-RT-R | CTATCAGGAGATTGGGCAGGTGCTTCAGCC |
| cenH-qPCR-F | GCTAAGATCGATTGGTGACG |
| cenH-qPCR-R | AAGTTCACTGTTCTTATACACTGG |
| mat s1-F | GCTAAATCGAAGGATTAAACAGTATCTTCC |
| mat s1-R | ACGAATTTGGCAGACTAGCCAC |
| mat s2-F | CATATCAATTACAAACATATTGTTTGCTGC |
| mat s2-R | GCTTCATGTTAAGTTGATTGGTGTAATTAG |
| ade6-qPCR-F | ATGCTTATCCTACAACTGAGACC |
| ade6-qPCR-R | TGAATTGAGAAGGGAAGACGAG |
| SPCC320.03-qPCR-F | GCGTATGATTGGTTCTGGATAGCCG |
| SPCC320.03-qPCR-R | GGGGCATGAGCTCATACCCATG |
| tub1-qPCR-F | AACGCTTGGCCATGGAATACACG |
| tub1-qPCR-R | GAGAGGCGGTGATGGAAGAAACAAC |
| GFP-qPCR-F | CGAAAGATCCCAACGAAAAGAG |
| GFP-qPCR-R | TCCCAGCAGCTGTTACAAACTC |
